# An update on the mosquito fauna and mosquito-borne diseases distribution in Cameroon

**DOI:** 10.1186/s13071-021-04950-9

**Published:** 2021-10-11

**Authors:** Roland Bamou, Marie Paul Audrey Mayi, Borel Djiappi-Tchamen, Stella Mariette Nana-Ndjangwo, Elysée Nchoutpouen, Antony John Cornel, Parfait Awono-Ambene, Phillipe Parola, Timoléon Tchuinkam, Christophe Antonio-Nkondjio

**Affiliations:** 1 Vector Borne Diseases Laboratory of the Biology and Applied Ecology Research Unit (VBID-URBEA), Department of Animal Biology, Faculty of Science of the University of Dschang, Dschang, Cameroon; 2 Organisation de Coordination pour la lutte Contre les Endémies en Afrique Centrale (OCEAC), Yaoundé, Cameroon; 3 Aix Marseille Univ, IRD, SSA, AP-HM, UMR Vecteurs-Infections Tropicales et Méditerranéennes (VITROME), Marseille, France; 4 IHU Méditerranée Infection, Marseille, France; 5 Laboratoire de Parasitologie et d’écologie, Université de Yaoundé 1, Yaoundé, Cameroun; 6 Department of Entomology and Nematology, Mosquito Control Research Laboratory, University of California, Davis, California USA; 7 Vector Biology Liverpool School of Tropical Medicine, Liverpool, UK

**Keywords:** Mosquito, Biodiversity, Distribution, Mosquito-borne diseases, Malaria, Lymphatic filariasis, Arboviruses, Cameroon

## Abstract

The expansion of mosquito-borne diseases such as dengue, yellow fever, and chikungunya in the past 15 years has ignited the need for active surveillance of common and neglected mosquito-borne infectious diseases. The surveillance should be designed to detect diseases and to provide relevant field-based data for developing and implementing effective control measures to prevent outbreaks before significant public health consequences can occur. Mosquitoes are important vectors of human and animal pathogens, and knowledge on their biodiversity and distribution in the Afrotropical region is needed for the development of evidence-based vector control strategies. Following a comprehensive literature search, an inventory of the diversity and distribution of mosquitoes as well as the different mosquito-borne diseases found in Cameroon was made. A total of 290 publications/reports and the mosquito catalogue website were consulted for the review. To date, about 307 species, four subspecies and one putative new species of Culicidae, comprising 60 species and one putative new species of *Anopheles*, 67 species and two subspecies of *Culex*, 77 species and one subspecies of *Aedes*, 31 species and one subspecies of *Eretmapodites*, two *Mansonia*, eight *Coquillettidia*, and 62 species with unknown medical and veterinary importance (*Toxorhynchites*, *Uranotaenia*, *Mimomyia*, *Malaya*, *Hodgesia*, *Ficalbia*, *Orthopodomyia*, *Aedeomyia*, and *Culiseta* and *Lutzia*) have been collected in Cameroon. Multiple mosquito species implicated in the transmission of pathogens within *Anopheles*, *Culex*, *Aedes*, *Eretmapodites*, *Mansonia*, and *Coquillettidia* have been reported in Cameroon. Furthermore, the presence of 26 human and zoonotic arboviral diseases, one helminthic disease, and two protozoal diseases has been reported. Information on the bionomics, taxonomy, and distribution of mosquito species will be useful for the development of integrated vector management programmes for the surveillance and elimination of mosquito-borne diseases in Cameroon.

**Supplementary Information:**

The online version contains supplementary material available at 10.1186/s13071-021-04950-9.

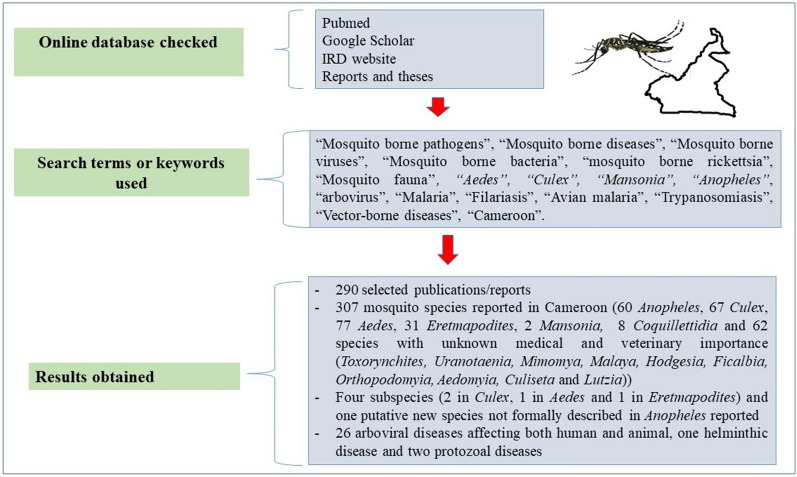

## Background

The emergence of mosquito-borne diseases worldwide such as dengue, yellow fever, and chikungunya has rekindled the interest and need to establish active surveillance programmes of common and neglected insect-borne human infectious diseases. Early detection of infected arthropods can facilitate control responses to prevent the spread of outbreaks with significant public health consequences. According to the World Health Organization (WHO), mosquito-borne diseases account for about 17% of the total burden of all infectious diseases [1]. Mosquitoes are blood-sucking dipterans belonging to the Culicidae family, with 3583 valid species currently described worldwide [2]. In this publication, we used the composite *Aedes* valid species list, where species are classified within 41 genera and 44 subgenera [2]. The genus *Aedes* has the highest number of species, with 33 species of uncertain subgeneric status and 900 classified into 72 subgenera. The *Culex* genus is the second largest, with 763 species described within 26 subgenera, whereas the genus *Anopheles* comprises seven subgenera with about 460 species [3–5].

Mosquitoes transmit a variety of diseases of medical or veterinary importance such as malaria, filariasis, encephalitis, yellow fever, dengue, Rift Valley fever, and other diseases [6–16]. Malaria is the most commonly known mosquito-borne disease in the world, and close to 50% of the world’s population live in areas at risk of infection [17]. In 2019, malaria was responsible for about 210 million febrile cases and 405,000 deaths, with more than 90% of cases occurring in sub-Saharan Africa [17]. Some arboviral diseases are also spreading worldwide, especially dengue, with 40% of the population infected globally and about 3.9 billion people living in areas at risk of contracting the disease, whereas about 10 years ago, less than 13% of the world’s population and about 2.5 billion people were at risk [18]. The remaining arboviral diseases are associated with severe symptoms or high lethality in humans (yellow fever), abortion (zika), or acute febrile symptoms associated with pain and fever (chikungunya) [19–21]. Apart from these well-known vector-borne diseases, mosquitoes are also involved in the transmission of a large variety of pathogens affecting both humans and animals, such as the West Nile virus (*Flaviviridae*, *Flavivirus*), Rift Valley fever (*Phenuiviridae*, *Phlebovirus*), Wesselsbron virus (*Flaviviridae*, *Flavivirus*), Middelburg virus (*Togaviridae, Alphavirus*), *Wuchereria bancrofti* (Spirurida, Onchocercidae), avian malaria, avian trypanosomiasis, avian filarial worms, and bacterial diseases [15, 19, 21–33]. Many of these diseases are less frequent in humans and mostly affect animals, and there are still insufficient data on their prevalence and distribution in different environments in the Afrotropical region.

Cameroon has a diverse mosquito fauna with over 300 mosquito species thus far recorded in the country. The bionomics and distribution of Anopheline species have been extensively studied in Cameroon, and in many parts of the country, malaria transmission dynamics are complex and stable because of multiple vector species occurring in the same locality [7, 16, 22–25, 27, 32, 34–48]. The distribution and bionomics of almost all the remaining mosquito genera are still not well documented across Cameroon. Moreover, the implication of these mosquito species in diseases or pathogen transmission is not well documented. The present review provides a synopsis of current information on the bionomics, distribution, and role in disease transmission of the mosquito fauna in Cameroon.

## Retrieval of information

We followed a similar methodology as Azari-Hamidian et al. [49] for searching the literature. Briefly, published reference documents on medical and veterinary entomology were reviewed to collect information on mosquitoes, diseases, and pathogens [50–53]. An online search of scientific papers on mosquito-borne diseases using different search terms was undertaken using online bibliographic databases such as PubMed, Google, and Google Scholar. A combination of the following search terms were used to select publications on mosquito-borne diseases or mosquito species: “mosquito borne pathogens”, “mosquito borne diseases”, “mosquito borne viruses”, “mosquito borne bacteria”, “mosquito borne rickettsia”, “mosquito fauna’’, mosquito genera including “*Aedes*”, “*Culex*”, “*Mansonia”*, “*Anopheles*”, “arbovirus”, “malaria”, “filariasis”, “avian malaria”, “trypanosomiasis”, “vector-borne diseases”, and “Cameroon”. In addition to the online bibliographic databases searched, data were also extracted from reports and theses.

## Presentation of Cameroon

Cameroon (1°40–13°05N, 8°30–16°10E) is in Central Africa, along the Guinea Gulf and covers a surface area of 475,000 km^2^ with a coastal border of about 400 km along the Atlantic Ocean. The country has a population of about 25 million inhabitants [54]. The mean density of the population is 49.5 person/km^2^. There is an increase in migration of the population from rural to urban settings, and it is estimated that about 2% of the population moves from rural to urban settings yearly. Neighbouring countries include Nigeria to the west, Chad to the north and east, Central African Republic to the east, and Democratic Republic of the Congo, Gabon, and Equatorial Guinea to the south [54, 55].

The country is divided into 10 administrative units called regions and has highly heterogeneous landscapes, with mountain peaks approaching 4000 m (Mount Cameroon), plains, and plateaus. Ecological domains with particular associated climatic conditions result in a high variety of ecological settings across the country [7, 56]. Annual rainfall varies from 400 mm/year in the Sahelian zone to 10,000 mm/year at the foot of Mount Cameroon. The average temperature varies between 18 °C and 28 °C [57].

## Mosquito-borne diseases and pathogens circulating in Cameroon

A high variety of mosquito-borne pathogens responsible for diseases in humans and animals have been reported to circulate in Cameroon. These include parasites, filarial worms, and arboviruses (Table 1).

**Table. 1:** Distribution of mosquito-borne diseases, pathogens, main vectors, and related hosts in Cameroon

Diseases	Pathogens	Main vectors	Hosts detected infected	Distribution sites	References
Human malaria	*P. falciparum*, *P. ovale*, *P. malariae*, *P. vivax*	*Anopheles gambiae*, *An. funestus*, *An. coluzzii*, *An. arabiensis*	Humans	Country wide	[6, 7, 16, 23, 25, 32, 35, 38, 45, 101, 102, 117, 118]
Primate malaria	*P. adleri*, *P. blacklocki*, *P. praefalciparum*, *P. reichnowii*, *P. gaboni*, *P. billcollinsi*	*An. moucheti*	Great apes such as gorillas, chimpanzees	South forest region	[64–66]
Avian malaria	*Plasmodium* spp., *Haemoproteus* spp.	*Culex* spp., *Mansonia* spp*.*, *Coquillettidia* spp.	Birds	South-West (Nguti), South (Ndibi, Mvia, Koto)	[15, 29, 74]
Lymphatic filariasis	*Wuchereria bancrofti*	*Anopheles* spp., *Mansonia* spp., *Culex* spp.	Humans	Northern Cameroon	[201]
Dengue fever	*Dengue virus* (serotype 1–4)	*Aedes albopictus*, *Ae. aegypti*	Humans, animals (squirrels, monkeys, parrots, héron, calao)	Douala, Yaoundé, Kaele, Bankim, Ntui, Edea, Buea, Foumban, Dschang, Bafia, Bangangte, Kribi, Garoua,	[28, 81, 86, 87, 93, 97, 98, 100, 158, 159]
Chikungunya	*Chikungunya virus* 1 genotype (East Central South African genotype)	*Ae. albopictus*, *Ae. aegypti*	Humans, animals (mammals, birds)	Kumbo, Buea, Tiko, Douala, Yaoundé	[28, 86, 87, 89, 90, 94, 109]
Yellow fever	*Yellow fever virus*	*Ae. albopictus*, *Ae. aegypti*	Humans, animals (mammals, birds)	Kumbo, Buea, Ayos, Mora, Bertoua, Batouri, Mokolo	[28, 84, 86, 87, 89, 90, 201]
Zika	*Zika virus*	*Ae. albopictus*, *Ae. aegypti*	Humans, animal (migrant birds, francolin, calao, rapace, Passeriformes, pigeons, mammals)	Garoua, Maroua, Ngaoundere, Buea, Bertoua, Yaoundé, Douala	[96]
Rift Valley fever	*Rift Valley fever virus*	*Aedes* spp., *Culex* spp.	Humans, goats, sheep, cattle, gazelles, buffalo	Countrywide (in all regions of the country)	[19, 21, 30, 92]
Other viruses	*Tiko virus*, *Kumba virus*, *Semlinki virus*, *Okola virus*, *Tahina virus*, *Onyong nyong virus*, *Ilesha virus*, *MIDV*, *NTAV*, *WESV*, *Bunyamwera virus*, *Eret virus*, *Silbu virus*, *Tatanguine virus*, *Osuntu virus*, *Uganda virus*, *Sindbis virus*, *Nkolbisson virus*, *SPOV*	*Culex* spp., *Mansonia* spp., *Coquillettidia* spp., *Eretmapodites* spp.	Humans, animals (mammals, birds)	Kumba, Obout, Buea, Ebogo, Ototomo, Mbalmayo, Akonolinga, Nkolbisson, Okola, Ofoumselek, Yaoundé	[28, 84, 86–90]

### Malaria

Malaria is a parasitic disease that affects both humans and animals, including birds and great apes (gorillas and chimpanzees). Human malaria is endemic across the country with differences in its prevalence between regions [58]. Cameroon is among the 11 countries most affected by malaria in the world. In 2018, about 2,133,523 malaria cases and 3263 associated deaths were reported in health facilities in Cameroon [58]. Currently, it is estimated that 28% of the population suffers yearly from malaria attacks, and the prevalence of the disease increased by 4% in 2019 as compared to 2018 [58]. The East, Adamaoua, and Central regions exhibit the highest morbidity (about 162, 142, and 120 cases for 1000 inhabitants, respectively), while the overall mean of morbidity in the country is 102 cases for 1000 inhabitants [58]. Four *Plasmodium* (Haemosporidia, Plasmodiidae) species infecting humans have been reported in the country, namely *P. falciparum*, *P. vivax*, *P. ovale*, and *P. malariae* [7, 59–63]. Six *Plasmodium* (*Laverania*) spp. infecting primates have also been reported, including *P. reichnowii*, *P. gaboni*, and *P. billcollinsi* in chimpanzees and *P. adleri*, *P. blacklocki*, and *P. praefalciparum* in gorillas [64–66]. Anopheline species such as *An. moucheti* are considered to be a possible bridge vector between humans and apes [67]. *Plasmodium falciparum*-like parasites infecting great apes in southern Cameroon were not found to represent a recurrent source for human malaria [68].

Avian malaria caused by several *Plasmodium* and *Haemoproteus* (Haemosporida, Haemoproteidae) species occur in Cameroon [15]. Avian malaria affects a wide range of birds globally and is responsible for high lethality in bird populations in areas where previous exposure to the parasites was limited or absent [69]. Recent studies in the South-West region (Nguti) of Cameroon reported a high prevalence of avian malaria parasites in all bird families (16.1% for *Plasmodium* infections and 11.6% for *Haemoproteus* infections) [15]. Unlike human *Plasmodium*, whose vectors are found only among anophelines, species within the *Culex* and *Aedes* are considered as the primary vectors of avian *Plasmodium* species. Other species within the genera *Culiseta* (Diptera, Culicidae), *Anopheles*, *Mansonia*, *Aedeomyia*, *Uranotaenia*, and *Coquillettidia* have been implicated in the transmission of avian *Plasmodium* [29, 70–73]. Studies conducted so far in Cameroon have identified *Culex* spp., *Coquillettidia* spp., and *Mansonia* spp. as vectors of avian malaria [29, 74] (Table 1). Studies on avian malaria in Cameroon are still at the exploratory phase, and there is still much more to investigate to unveil the complexities of transmission, distribution, and epidemiology of avian malaria.

### Filariasis

Lymphatic filariasis (LF) is a neglected tropical disease that is targeted for elimination by 2030 [75]. It is caused by *W. bancrofti*, *Brugia malayi*, and *B. timori* (Spirurida, Onchocercidae) and is transmitted by *Culex*, *Mansonia*, and *Anopheles* mosquitoes [76]. In West Africa, the dominant vectors are *Anopheles gambiae* sensu lato and *An. funestus*, whereas in East Africa, it is transmitted by *Cx. quinquefasciatus* [75]. LF causes substantial morbidity and disabilities which can lead to social exclusion. The disease is on the decline in Cameroon due to the intensification of mass drug administration (MDA) campaigns across the country using the drugs albendazole and ivermectin [75]. A recent study conducted in 31 health districts of four endemic regions of the south of the country (central, east, south, and littoral) failed to find any cases of *W. bancrofti* infection [77]. Due to cross-reaction of the LF diagnostic test (filariasis test strip, FTS) with *Loa loa* (Spirurida, Filaridae), a microfilaria which is endemic in the southern part of the country, false positives are increasingly reported, and it is obvious that appropriate diagnostic tools avoiding false positive detections are needed to guide disease elimination efforts in the country [77, 78]. With the intensification of travel and population migrations between East and West Africa and within regions, it is possible that cases could be imported from other endemic settings which will make *W. bancrofti* filariasis elimination in Cameroon more challenging.

### Arboviruses

**Fig. 1:**
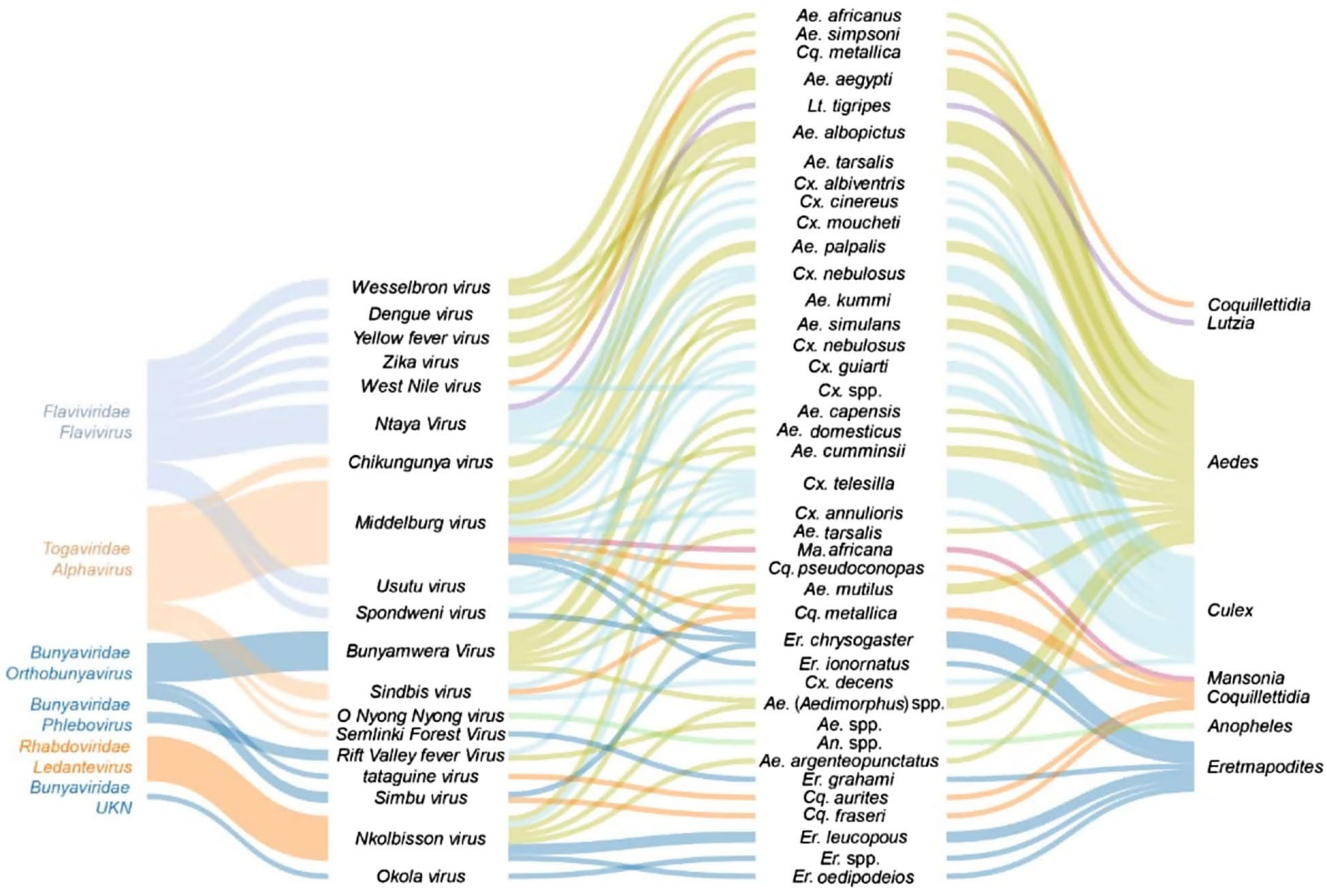
Sankey diagram showing relationship between arbovirus (family, species) detected in Cameroon and mosquito vectors (species and genera). The height of the bars represents the relative abundance of the groups within each network level

Arboviral diseases constitute a growing international public health threat, especially due to the absence of functional vaccines for some of the diseases, therapeutic drugs, and effective vector control programmes [79]. In Cameroon, different arboviruses have been reported in febrile and non-febrile patients in different localities in the country [21, 80–82]. The most prevalent include dengue, chikungunya, yellow fever, zika, and Rift Valley fever viruses [19, 21, 30, 83–91]. To date, at least 26 different arbovirus diseases belonging to five families have been reported in Cameroon (see Table 1) [8, 21, 30, 31, 81, 84, 92, 93]. Among them, 18 arboviruses have been detected in humans [86, 87, 94, 95], and 14 are transmitted by mosquitoes (*Aedes*, *Culex*, *Eretmapodites*, *Mansonia*, and *Anopheles*) (Fig. 1). Arboviruses detected most frequently in Cameroon include chikungunya [28, 94], O’nyong nyong, Sindbis [87], Spondweni, Middleburg, Semlinki forest [28, 86], zika [86, 96], Tahyna [87], and dengue viruses [81, 93, 95, 97]. Dengue and chikungunya viruses have been reported in major cities such as Yaoundé, Douala, and Garoua, and in some rural settings [94, 98, 99]. Rift Valley fever is less common in humans despite evidence of infections in animals [21, 30, 92] with prevalence of IgG in blood samples varying from 3.4–12.3% in goats and sheep [30, 56], 9.3–13.5% in cattle [30, 56], and 12.4% in humans [21]. In general though, arboviral human and animal screenings have just been limited to a few ecological settings in Cameroon. Evidence of Rift Valley fever and Crime Congo haemorrhagic fever virus infections among pygmies in the east region of Cameroon have been reported [21], and it would not be surprising that these diseases could be more widely distributed across the country because of the presence of multiple competent mosquito vector species across the country [31, 100] (Table 1). Rift Valley fever virus cases in humans were reported in the littoral region (Nkongssamba) [80] and in the east region (Lomié, Missok, and Mindoumou) [21]. Arbovirus circulation and distribution across the country is still not well documented and deserves further attention to discern risks of outbreaks.

## Mosquito fauna of Cameroon: distribution, bionomics, and epidemiological role

Multiple mosquito-collecting surveys conducted since the 1940s have overall found a high diversity of mosquito species in Cameroon [15, 33, 48, 53, 84, 90, 101–111]. In total, 307 species, four subspecies and one putative new species within 16 genera have been recorded in the country so far. The distribution of major species with medico-veterinary importance is found in Additional file 1: Figure S1, Additional file 2: Figure S2, Additional file 3: Figure S3, and Additional file 4: Figure S4.

### *Anopheles* species

The genus *Anopheles* is the most important, with several species playing major roles in the transmission of malaria and lymphatic filariasis to humans. This genus is composed of seven subgenera with about 460 species [3–5]. According to recent records, 60 species and one putative new species have been reported in Cameroon [7, 112–116] (Additional file 5: Table S1). Their distribution varies between ecological zones (see Additional file 1: Figure S1). Species such as *An. gambiae*, *An. arabiensis*, *An. coluzzii*, *An. funestus*, *An. nili*, and *An. moucheti* known for their high anthropophilic behaviour are considered major vectors of malaria in the country [6, 7, 16, 25, 33–35, 42, 101, 102, 117–120]. Other species involved in malaria transmission include *An. carnevalei*, *An. coustani*, *An. hancocki*, *An. leesoni*, *An. marshallii*, *An. melas*, *An. paludis*, *An. pharoensis*, *An. ovengensis*, *An. wellcomei*, *An. rufipes*, and *An. ziemanni* [6, 16, 24, 27, 32, 34, 45, 48, 102]. *Anopheles* mosquitoes are also known to transmit diseases such as O’Nyong Nyong (family *Flaviviridae*) and lymphatic filariasis [75], but studies conducted so far in Cameroon specifically have not incriminated anopheline in the transmission of these diseases.

According to WHO, indoor residual spraying (IRS) and long-lasting insecticidal nets (LLINs) are the cornerstone in the fight against malaria vectors in most endemic countries. In Cameroon, LLINs are the only tools used for malaria vector control by the National Malaria Control Program [58, 121]. Despite successes gained so far in the fight against malaria vectors, vector control faces diverse challenges due to multiple vector species, changes in feeding and biting behaviour of anopheline, and the rapid expansion of insecticide resistance in the main malaria vectors [22, 102, 113, 122]. The rapid expansion of insecticide resistance in Cameroon seems to be driven by the increase use of insecticides in both public health and agriculture [123]. A recent review on insecticide resistance evolution in Cameroon [22] indicated high resistance to almost all insecticide classes (pyrethroids, carbamates, organochlorines, and organophosphates) driven by both target site- and metabolic-based mechanisms [7, 22, 113, 124–126]. In addition to insecticide resistance, behavioural changes could also affect the efficacy of control interventions, but these have so far received limited attention [102].

### *Culex* species

*Culex* is a genus comprising more than 700 species worldwide belonging to 26 subgenera [127]. So far, 67 species and two subspecies of *Culex* have been collected in Cameroon [15, 74, 84, 104, 109, 128–131] (Additional file 5: Table S2). It is likely that the number of species known from the Cameroon fauna is underestimated due to difficulties associated with their identification, especially with species that differ only in male genitalia structure within the *Culiciomyia* and *Eumelanomyia* subgenera. *Culex quinquefasciatus*, *Cx. antennatus*, and *Cx. duttoni* appear to be the most common in urban settings [104, 105, 128, 130], but the general distribution of species of this genus varies with collection sites or region of the country (Additional file 2: Figure S2).

Immature stages of *Culex* are found in different types of habitats. In forests, *Culex* larvae are typically found in rock pools, tree-rot holes, river ditches, forest pools, leaf axils, crab holes, and epiphyte plant leaf axils [53, 104, 132]. In urban settings, *Culex* larvae are found in a variety of habitats including catch basins, storm drains, temporary vernal habitats between houses, septic tanks and open sewage systems rich of organic matters, road-side ditches, and in artificial containers such as rainwater barrels, tires, and bottles [130, 133]. The large adaptive capacity of some *Culex* mosquito species has facilitated their spread across the world. Some *Culex*, especially the more opportunistic blood-feeding species that feed on both humans and animals, are a nuisance and serve as arboviral bridge vectors in urban settings [134, 135].

*Culex* species are involved in the transmission of arboviruses such as West Nile, Rift Valley [136–139], Japanese encephalitis, St. Louis encephalitis, and Western and Eastern equine encephalitis viruses [8], *W. bancrofti*, and *Dirofilaria immitis* [140–142]. Species such as *Cx. neavei*, *Cx. poicilipes*, *Cx. perfidiosus*, *Cx. guiarti*, *Cx. vansomereni*, and *Cx. annulioris* have been reported to be involved in the transmission of avian malaria in Cameroon [74]. Other species such as *Cx. albiventris*, *Cx. nebulosus*, and *Culex telesilla* have been implicated in the transmission of Ntaya, Middleburg, West Nile, and Spondweni viruses [84] (Fig. 1, Table 1, and Additional file 5: Table S2).

Few studies have investigated the impact of control interventions on *Culex* mosquitoes in Cameroon [143, 144]. Some indoor biting *Culex* found in sympatry with *Anopheles*, particularly in urban settings, could be affected by LLIN and IRS control measures. However, one study indicated a low impact of these measures on *Culex* mosquito abundance [130]. As a result of intense selection pressure induced by insecticides used in agriculture and public health, *Culex* have likely become increasingly resistant to most chemicals used for vector control, such as permethrin, deltamethrin, DDT, and bendiocarb [130]. A larval control trial using *Bacillus sphaericus* (*Bacillales*, *Bacillaceae*), now named *Lysinibacillus sphaericus*, failed to control *Cx. quinquefasciatus* in the city of Maroua, Cameroon [144]. Larvivorous fishes such as *Poecilia reticulata* (Cyprinodontiformes, Poeciliidae) are frequently used in lakes and permanent water collection in urban settings to control *Cx. quinquefasciatus* [145].

Because little attention is generally paid to *Culex* in Africa, data on species diversity, bionomics, distribution, and the vectorial role of species within this genus remain incomplete in Cameroon. With the increased adaptive capacity and rapid expansion of insecticide resistance in some species, integrated strategies to control both *Culex* and *Anopheles* should be promoted.

### *Aedes* species

**Fig. 2:**
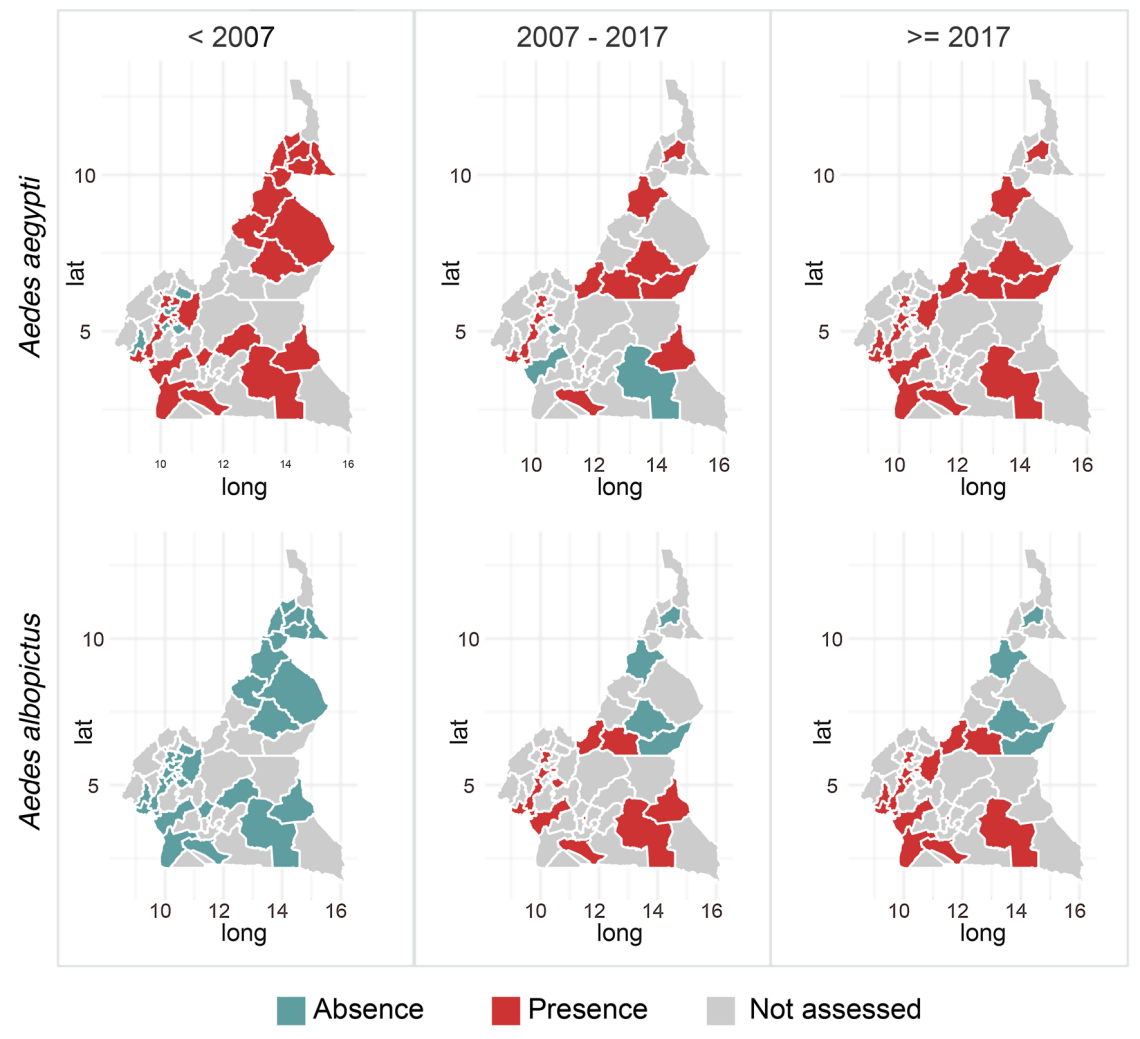
Distribution of major vector *Aedes* species in Cameroon according to year

The genus has the highest number of species worldwide with up to 1256 species [5, 146]. A total of about 77 species and one subspecies have been reported in Cameroon (Additional file 5: Table S3). There is still an uncertainty on the true number of *Aedes* species distributed in Cameroon [147]. Four species are by far the most mentioned or studied in the country. These include *Ae. aegypti*, *Ae. albopictus*, *Ae. africanus*, and *Aedes simpsoni* [56, 148]. These species are widely distributed across the country (Additional file 3: Figure S3). *Aedes albopictus* was reported for the first time in Cameroon in 2001 [149] and is now abundant in urban settings, whereas *Ae. aegypti* predominates in suburban and rural areas. Since the first report of *Ae. albopictus*, it has extended its distribution range to almost all the country except in the north and far-north regions (Fig. 2). *Aedes africanus* and *Ae. simpsoni* are abundant in rural and forest settings (see Additional file 3: Figure S3) [15, 41, 74, 84, 103, 104, 109, 130].

In rural settings, *Ae. aegypti*, *Ae. albopictus*, and *Ae. simpsoni* larvae are mainly found in containers used to store water, but also in tree holes, cocoa pods, snail shells, and fallen large leaves holding water on the ground. In urban areas, larvae of these species are most often found in artificial containers such as tires, flower pots, broken bottles, plastic cups, tanks, cinder blocks, and abandoned containers [56, 149–151]. The eggs of *Aedes* are resistant to desiccation for several months [152], allowing them to survive the dry season [150], and the females usually bite during the day in shaded areas, with peak activity in the morning and late in the afternoon [149].

Multiple arboviruses have been isolated from different *Aedes* mosquito species in Cameroon, highlighting their role as epidemiologically important (Table 1 and Additional file 5: Table S3). *Aedes aegypti* and *Ae. albopictus* are the most well-known vectors of arboviruses such as dengue, chikungunya, yellow fever, zika, and West Nile viruses [153–157]. Cases of dengue, chikungunya, and yellow fever viruses have been frequently reported in Cameroon [158, 159] with cases of yellow fever mostly documented in rural settings and dengue and chikungunya more prevalent in urban settings. The current expansion of *Ae. albopictus* in Cameroon [148] could result in increased risk of arbovirus disease transmission across the country, which deserves further investigations.

Knowledge of the bionomics of almost all *Aedes* species, except a little known for the ones mentioned above, is severely lacking in Cameroon. Changes in the environment due to deforestation, urbanisation, and agricultural practices will likely affect dynamics of “*Aedes*-borne” arboviral diseases which may put more people at risk of contracting diseases such as Rift Valley fever.

There is no specific intervention targeting *Aedes* populations in Cameroon, and the existing risk of arboviral infection outbreaks due to *Aedes* is unknown. The current national vector control strategies (LLINs) target only the indoor biting species and species that feed predominantly at night, and most *Aedes* species bite outdoors and during the day and at sunset. Different studies suggest increased tolerance of *Aedes* mosquito populations to DDT, permethrin, deltamethrin, and propoxur [150, 151]. Insecticide resistance, which affects both *Ae. aegypti* and *Ae. albopictus*, is still emerging and limited to urban settings. Mechanisms involved in *Aedes* species resistance in Cameroon have not been fully investigated [150, 151]. Controlling mosquito larvae with either *B. sphaericus* and/or *B. thuringiensis* (*Bacillales*, *Bacillaceae*) could be useful for *Aedes* control, although challenging, because of the diversity and numbers of small habitats in which immatures develop.

### Other mosquito genera of medical and veterinary importance

#### *Eretmapodites* species

The genus *Eretmapodites* consists of 48 species, three of which includes nominotypical and one subspecies [2]. All *Eretmapodites* species are restricted to the African continent [160]. Most *Eretmapodites* species occur in Cameroon; in fact, 31 species and one subspecies are recorded in this country (Additional file 5: Table S4). *Eretmapodites* species found so far in Cameroon were collected in the West region (Dschang), Central region (Yaoundé), South region (Mvina, Dja et lobo), and South-West region (Nguti in Talangaye forest) [15, 31, 74, 83, 84, 103, 104, 111] (see Additional file 4: Figure S4). There could be more than the 32 species present in Cameroon because the methods used to capture mosquitoes in general mosquito surveys and traps do not capture *Eretmapodites* very well. Furthermore, most identifications of *Eretmapodites* are limited to the generic level because of the similarities in species morphologically, and most species can only be reliably identified based on male genitalia, requiring dissection and careful mounting on slides for examination [160, 161].

Most *Eretmapodites* species occur in pristine thickly forested areas, with a few adapted to small riverine and mountainous wooded enclaves in savanna regions. Very little is known about the bionomics of *Eretmapodites*, but they seem to be quite opportunistic blood feeders with many species, including the *Er. chrysogaster* group and *Er. parvipluma*, reported to feed on humans during the day and crepuscular periods [160].

The few *Eretmapodites* species that have adapted to more open rural environments near human dwellings, such as *Er. chrysogaster*, are typically known as “container breeders”. For example, immatures of the *Er. chrysogaster* group, which are usually abundant in large fallen leaves that hold water in forest floors, can be found in artificial containers such as discarded tins and pots, but only when these are in shade and contain water strongly tinged by decaying vegetable matter. In pristine forests, it is believed that *Eretmapodites* mainly select habitats such as epiphyte plant leaf axils, fallen fruit husks and leaves, and empty snail shells as oviposition sites [162]. Larvae of some species are facultative predators and feed on culicine larvae (*Ae. simpsoni*, *Ur. ornata musarum*, and *Cx. nebulosus*) which are also common in plant axils [163].

*Eretmapodites* spp. are known to transmit yellow fever to rhesus monkeys in laboratory settings [164]. Several virus strains such as Rift Valley [165], Spondweni [166], and Semliki forest viruses have been isolated from *Eretmapodites* species. In Cameroon, *Er. grahami* was reported to be infected by the Semliki forest virus [88]. *Eretmapodites chrysogaster* was found infected with the Nkolbisson and Simbu viruses [167]. Other viruses found within *Eretmapodites* include Nyando virus [168], Chikungunya virus [169], Okola, Middleburg, and Bunyamwere viruses [170], and Ntaya and Spondweni viruses [8, 171].

Due to their low epidemiological importance and local nuisance, there are no official control measures against these potential vectors in Cameroon. However, because they bite during the day and outdoors, the use of repellents could be effective against these mosquitoes, especially in areas where they can be a nuisance such as in banana plantations.

#### *Coquillettidia* species

Eight *Coquillettidia* species have been reported in Cameroon (Additional file 5: Table S5) [83, 102, 103, 130, 161]. These include *Cq. pseudoconopas*, *Cq. maculipennis*, *Cq. aurites*, *Cq. annettii*, *Cq. fraseri*, *Cq. metallica*, and *Cq. versicolor* in the West (Dschang), Central (Yaounde), and South regions [29, 31, 74, 84, 103]. The bionomics of African *Coquillettidia* species is poorly known. The immature stages attach to stems of various plants, in shallow water, marshes, swamps, ponds, lakes, and seepage. Adults are exceptionally active in both day and night, and females feed on both animal and human blood [2, 164]. Their distribution is presented in Additional file 4: Figure S4.

Some *Coquillettidia* species have been implicated in the transmission of protozoans and viruses to humans and animals [8, 29, 74, 172]. In Cameroon, *Cq. pseudoconopas*, *Cq. aurites*, and *Cq. metallica* have been implicated in the transmission of avian malaria [29]. *Coquillettidia* species such as *Cq. perturbans*, a species found in the USA, was found to be a competent vector of West Nile virus in the laboratory [173]. Moreover, *Cq. maculipennis* and *Cq. versicolor* are potential vectors of chikungunya virus and Rift Valley fever virus, respectively [174].

Due to the minimal nuisance and low known disease importance, there have so far not been any interventions targeting African species of *Coquillettidia*. However, *Coquillettidia* mosquitoes can be controlled by removal of hydrophytic vegetation because of their reliance on submerged vegetation for gaseous exchange. In the USA, where these mosquitoes are important vectors of disease for humans and animals, control strategies were first based on the aerial spraying of insecticides (Arosurf^®^ MSF and fenitrothion) on flying adults or ponds [175, 176] and traps [177, 178]. Studies conducted so far have shown that adult management remains poorly effective or not easily applicable. Moreover, most conventional methods for controlling the immature aquatic stages of other mosquitoes are ineffective against this group because they do not rise to the water surface until eclosion and often occur in densely vegetated water bodies, which minimises effective exposure to larvicides [179, 180]. Acceptable control has been obtained using an insect growth regulator such as methoprene to control *Cq. perturbans* in place of *B. thuringiensis* and temephos (organophosphate) [181]. Another control method for this group is the removal of vegetation in rivers, but this can lead to ecological disturbance or imbalance.

#### *Mansonia* species

In Cameroon, *Mansonia africana* and *Ma. uniformis* are quite prolific in areas near water supporting hydrophytic vegetation [74, 84, 111, 130]. Both species are distributed countrywide and are mainly found in areas with slow-flowing rivers and marshlands favourable for immature stage development (Additional file 4: Figure S4 and Additional file 5: Table S6). *Mansonia africana* and *Ma. uniformis* have been reported in the country since 1937 [111] (Additional file 5: Table S6). *Mansonia uniformis* is sometimes considered the most abundant species in Cameroon and in Central Africa [122]. *Mansonia* mosquitoes are active mostly at dusk and dawn but sometimes bite at night indoors and mostly outdoors [122, 135, 182] and occasionally are found resting indoors [122]. *Mansonia* feed on both humans and animals such as cattle, fowl, goats, and dogs [183, 184]. *Mansonia* larvae are found in rivers supporting thick vegetation along the edges, and in swamps to which they attach by piercing their siphons to breathe from submerged plant stems (especially *Pistia*) [185, 186].

*Mansonia* species are involved in the transmission of arboviruses [174] and lymphatic filariasis caused by *W. bancrofti* [187–189], *B. malayi*, and *B. timori* [190]. Several arboviruses including Banzi, Bunyamwera, chikungunya, Rift Valley fever, Sindbis, Spondweni, and Wesselsbron viruses have been reported to be transmitted by *Mansonia* species [174]. Studies conducted so far in Cameroon were unable to link the transmission of *W. bancrofti* to *Mansonia* species. *Mansonia uniformis* has been implicated in the transmission of avian malaria in forested areas of Cameroon [74] (Additional file 5: Table S6).

Occasional blood-feeding at night and indoor resting habits of both *Mansonia* species means they can be controlled by IRS and LLINs. Additional control tools such as repellents, coils, and screens on windows could be used for controlling this mosquito species. There are still no data on the susceptibility of this species to insecticides in Cameroon.

### Mosquito species of unknown or minimal medical and veterinary importance

A list of 62 mosquito species belonging to other genera is provided in Additional file 5: Table S7. *Toxorhynchites* and *Malaya* are non-hematophagous and therefore of no consequence as disease vectors. *Toxorhynchites* larvae are predaceous, and they have been proposed as a biological means of control for major container disease vector species such as *Ae. aegypti*, although field attempts to do so have not been successful [191].

*Mimomyia* and *Uranotaenia* species are mostly herpetophilic and seldom feed on the blood of mammals, but are likely responsible for transmission of anuran intracellular apicomplexans and microfilariae, as found in South Africa [192–194].

Due to a lack of isolation and attempts to do so, the genera *Hodgesia*, *Ficalbia*, *Orthopodomyia*, *Aedeomyia*, *Culiseta*, and *Lutzia* have thus not been implicated as disease vectors in Cameroon and Africa more generally, and their disease relationships are unknown [195].

## Conclusions

The present review provides information on the bionomics, distribution, and epidemiological role of mosquito species present in Cameroon. Even though multiple entomological surveys have been conducted in the country, only a few have investigated the diversity and distribution of non-malaria mosquito vectors, probably because of their perceived low medical and epidemiological importance at the national level. The review reveals a great diversity of the Cameroonian mosquito fauna, with over 300 species recorded. Among these, many species are vectors of human and animal pathogens. Cameroon has not yet developed a national strategy for integrated control of vector-borne diseases. There is still a huge shortage in well-trained entomologists able to identify non-*Anopheles* spp. In addition to training activities on basic entomology, courses on new molecular techniques are also needed to improve the capacity of technicians to properly identify and process all mosquito samples. The past decade has seen the development of new molecular tools and easy-to-use light technology such as matrix-assisted laser desorption/ionization-time of flight mass spectrometry (MALDI-TOF MS) which can significantly improve mosquito species identification [196–199], blood meal source detection [199], and pathogen detection [200]. Developing capacities in both new and classical techniques is paramount in driving efforts toward vector-borne disease elimination. Although increasing efforts have been made during the last decade for the elimination of diseases such as lymphatic filariasis and human African trypanosomiasis, most diseases are still prevalent in Cameroon and need additional efforts to achieve elimination targets. Involving communities in the removal of unnecessary water-holding containers and trash, and the use of improved irrigation practices could help ensure sustainability and the success of control interventions.

## Supplementary Information

**Additional file 1: Figure S1.** Leaflet map of main *Anopheles* species found in Cameroon.

**Additional file 2: Figure S2.** Leaflet map of main *Culex* species found in Cameroon.

**Additional file 3: Figure S3.** Leaflet map of main *Aedes* found in Cameroon.

**Additional file 4: Figure S4.** Leaflet map of other mosquito groups found in Cameroon.

**Additional file 5: Table S1.** *Anopheles* species composition, pathogens transmitted and control interventions in Cameroon. **Table S2.** *Culex* species composition, pathogens transmitted and control interventions in Cameroon. **Table S3.** *Aedes* species composition, pathogens transmitted and control interventions in Cameroon. **Table S4.** *Eretmapodites* species composition, pathogens transmitted and control interventions in Cameroon. **Table S5.** *Coquillettidia* species composition in Cameroon, pathogens transmitted and control interventions. **Table S6.** *Mansonia* species composition, pathogens transmitted and control interventions in Cameroon. **Table S7.** Mosquito species of unknown or minimal medical and veterinary importance: species composition, pathogens transmitted and control interventions in Cameroon.

## Data Availability

The references supporting the conclusions of this review are cited in the text, and data is also available in additional files.

## Abbreviations

LLINs: Long-lasting insecticidal nets
IRS: Indoor residual spraying
FTA: Filariasis test strip
LF: Lymphatic filariasis
MDA: Mass drug administration
MIDV: Middleburg virus
NTAV: Ntaya virus
WESV: Wesselsbron virus
CHIKV: Chikungunya virus
DENV: Dengue virus
YFV: Yellow fever virus
ZIKV: Zika virus
RVFV: Rift Valley fever
SPOV: Spondweni virus
NKOV: Nkolbisson virus
SIMV: Semlinki virus
MALDI-TOF MS: Matrix-assisted laser desorption/ionization-time of flight mass spectrometry

## References

[1] World Health Organization (2020). Evaluation of genetically modified mosquitoes for the control of vector-borne diseases.

[2] Harbach RE. Mosquito taxonomic inventory; 2013. http://mosquito-taxonomic-inventory.info/*.* Accessed 27 Apr 2021.

[3] Freitas LA, Russo CAM, Voloch CM, Mutaquiha OCF, Marques LP, Schrago CG (2015). Diversification of the genus *Anopheles* and a Neotropical clade from the late Cretaceous. PLoS ONE.

[4] Harbach R, Manguin S (2013). The phylogeny and classification of Anopheles. *Anopheles* mosquitoes-new insights into malaria vectors.

[5] Wilkerson RC, Linton Y-M, Fonseca DM, Schultz TR, Price DC, Strickman DA (2015). Making mosquito taxonomy useful: a stable classification of tribe Aedini that balances utility with current knowledge of evolutionary relationships. PLoS ONE.

[6] Antonio-nkondjio C, Kerah CH, Simard F, Awono-ambene P, Chouaibou M, Tchuinkam T (2006). Complexity of the malaria vectorial system in Cameroon: contribution of secondary vectors to malaria transmission. J Med Entomol.

[7] Antonio-Nkondjio C, Ndo C, Njiokou F, Bigoga JD, Awono-Ambene P, Etang J (2019). Review of malaria situation in Cameroon: technical viewpoint on challenges and prospects for disease elimination. Parasit Vectors.

[8] Braack L, de Almeida APG, Cornel AJ, Swanepoel R, de Jager C (2018). Mosquito-borne arboviruses of African origin: review of key viruses and vectors. Parasit Vectors.

[9] Dieme C, Bechah Y, Socolovschi C, Audoly G, Berenger J-M, Faye O (2015). Transmission potential of *Rickettsia felis* infection by *Anopheles gambiae* mosquitoes. Proc Natl Acad Sci.

[10] Johnson PD, Azuolas J, Lavender CJ, Wishart E, Stinear TP, Hayman JA (2007). *Mycobacterium ulcerans* in mosquitoes captured during outbreak of Buruli ulcer, southeastern Australia. Emerg Infect Dis.

[11] Kay BH, Farrow RA (2000). Mosquito (Diptera: Culicidae) dispersal: implications for the epidemiology of Japanese and Murray Valley Encephalitis viruses in Australia. J Med Entomol.

[12] Parola P, Musso D, Raoult D (2016). *Rickettsia felis*: the next mosquito-borne outbreak?. Lancet Infect Dis.

[13] Rodhain F (1985). Précis d’entomologie médicale et vétérinaire : notions d’épidémiologie des maladies à vecteurs.

[14] Singh H, Singh OP, Akhtar N, Sharma G, Sindhania A, Gupta N (2019). First report on the transmission of Zika virus by *Aedes* (Stegomyia) *aegypti* (L.) (Diptera: Culicidae) during the 2018 Zika outbreak in India. Acta Trop.

[15] Tchoumbou MA, Mayi MPA, Malange ENF, Foncha FD, Kowo C, Fru-cho J (2020). Effect of deforestation on prevalence of avian haemosporidian parasites and mosquito abundance in a tropical rainforest of Cameroon. Inter J Parasitol.

[16] Tchuinkam T, Simard F, Lélé-Defo E, Téné-Fossog B, Tateng-Ngouateu A, Antonio-Nkondjio C (2010). Bionomics of anopheline species and malaria transmission dynamics along an altitudinal transect in Western Cameroon. BMC Infect Dis.

[17] WHO. World malaria report. Geneva: World Health Organization; 2020 https://www.who.int/publications/i/item/9789240015791. Accessed 22 Dec 2020.

[18] WHO. Dengue guidelines for diagnosis, treatment, prevention and control, TDR for research on diseases and poverty, New edition; WHO; 2009. p. 160. https://apps.who.int/iris/handle/10665/44188. 23762963

[19] LeBreton M, Umlauf S, Djoko CF, Daszak P, Burke DS, Kwenkam PY (2006). Rift valley fever in goats. Cameroon Emerg Infect Dis.

[20] Maurice Y, Provost A (1969). Sondages sérologiques sur les arboviroses animales en Afrique centrale (peste équine, Blue Tongue, maladie de Wesselsbron, fièvre de la vallée du Rift). Rec Elev Méo Vet Pays Trop.

[21] Sadeuh-Mba SA, Wansi GMY, Demanou M, Gessain A, Njouom R (2018). Serological evidence of rift valley fever Phlebovirus and Crimean-congo hemorrhagic fever orthonairovirus infections among pygmies in the East region of Cameroon. Virol J.

[22] Antonio-Nkondjio C, Sonhafouo-Chiana N, Ngadjeu CS, Doumbe-Belisse P, Talipouo A, Djamouko-Djonkam L (2017). Review of the evolution of insecticide resistance in main malaria vectors in Cameroon from 1990 to 2017. Parasit Vectors.

[23] Atangana J, Bigoga JD, Patchoké S, Ndjemaï MNH, Tabue RN, Nem TE (2010). Anopheline fauna and malaria transmission in four ecologically distinct zones in Cameroon. Acta Trop.

[24] Awono-ambene HP, Kengne P, Simard F, Antonio-Nkondjio C, Fontenille D (2004). Description and bionomics of *Anopheles* (Cellia) *ovengensis* (Diptera: Culicidae), a new malaria vector species of the *Anopheles nili* group from South Cameroon. J Med Entomol.

[25] Ekoko WE, Awono-Ambene P, Bigoga J, Mandeng S, Piameu M, Nvondo N (2019). Patterns of anopheline feeding/resting behaviour and *Plasmodium* infections in North Cameroon, 2011–2014: implications for malaria control. Parasit Vectors.

[26] Elikwo MNF, Nota AD, Adele TM (2020). Effects of deforestation on avian parasitic co-infections in recaptured birds from an African tropical rainforest. Nano Tech Appl.

[27] Mbakop LR, Awono-Ambene PH, Mandeng SE, Ekoko WE, Fesuh BN, Antonio-Nkondjio C (2019). Malaria transmission around the Memve’ele hydroelectric dam in South Cameroon: a combined retrospective and prospective study, 2000–2016. Int J Environ Res Public Health.

[28] Ndip LM, Bouyer DH, Da Rosa APT, Titanji VPK, Tesh RB, Walker DH (2004). Acute spotted fever rickettsiosis among febrile patients, Cameroon. Emerg Infect Dis.

[29] Njabo KY, Cornel AJ, Sehgal RN, Loiseau C, Buermann W, Harrigan RJ (2009). *Coquillettidia* (Culicidae, Diptera) mosquitoes are natural vectors of avian malaria in Africa. Malar J.

[30] Rissmann M, Eiden M, Wade A, Poueme R, Abdoulkadiri S, Unger H (2017). Evidence for enzootic circulation of rift valley fever virus among livestock in Cameroon. Acta Trop.

[31] Salaun JJ, Rickenbach A, Brès P, Brottes H, Germain M, Eouzan J-P (1969). Les arbovirus isolés à partir de moustiques au Cameroun. Cahier OSTROM.

[32] Tabue RN, Nem T, Atangana J, Bigoga JD, Patchoke S, Tchouine F (2014). *Anopheles ziemanni* a locally important malaria vector in Ndop health district, North West region of Cameroon. Parasit Vectors.

[33] Tabue RN, Awono-Ambene P, Etang J, Atangana J, Antonio-Nkondjio C, Toto JC (2017). Role of *Anopheles (Cellia) rufipes* (Gough, 1910) and other local anophelines in human malaria transmission in the northern savannah of Cameroon: a cross-sectional survey. Parasit Vectors.

[34] Amvongo-Adjia N, Wirsiy EL, Riveron JM, Chounna Ndongmo WP, Enyong PA, Njiokou F (2018). Bionomics and vectorial role of anophelines in wetlands along the volcanic chain of Cameroon. Parasit Vectors.

[35] Awono-Ambene PH, Etang J, Antonio-Nkondjio C, Ndo C, Eyisap WE, Piameu MC (2018). The bionomics of the malaria vector *Anopheles rufipes* Gough, 1910 and its susceptibility to deltamethrin insecticide in North Cameroon. Parasit Vectors.

[36] Bigoga JD, Manga L, Titanji VP, Coetzee M, Leke RG (2007). Malaria vectors and transmission dynamics in coastal south-western Cameroon. Malar J.

[37] Bonnet S, Gouagna LC, Paul RE, Safeukui I, Meunier J-Y, Boudin C (2003). Estimation of malaria transmission from humans to mosquitoes in two neighbouring villages in south Cameroon: evaluation and comparison of several indices. Trans R SocTrop Med Hyg.

[38] Fondjo E, Robert V, Le Goff G, Toto J-C, Carnevale P (1992). Le paludisme urbain à Yaoundé (Cameroun): 2. Etude entomologique dans deux quartiers peu urbanisés. Bull Soc Pathol Exot.

[39] Hamon J, Adam JP, Grjebine A (1956). Observations sur la répartition et le comportement des anophèles de l’Afrique-Equatoriale Française, du Cameroun et de l’Afrique Occidentale. Bull World Health Organ.

[40] Languillon J, Mouchet J, Rivola E, Rateau J (1956). Epidemiology of malaria in the forest zone of the Cameroons. Med Trop.

[41] Le Goff G, Carnevale P, Fondjo E, Robert V (1997). Comparison of three sampling methods of man-biting anophelines in order to estimate the malaria transmission in a village of South Cameroon. Parasite.

[42] Mandeng SE, Awono-Ambene HP, Bigoga JD, Ekoko WE, Binyang J, Piameu M (2019). Spatial and temporal development of deltamethrin resistance in malaria vectors of the *Anopheles gambiae* complex from North Cameroon. PLoS ONE.

[43] Manga L, Robert V, Messi J, Desfontaine M, Carnevale P (1992). Le paludisme urbain à Yaoundé, Cameroun : 1. Etude entomologique dans deux quartiers centraux. Mém Soc Roy Entomol Belg.

[44] Manga L, Mbingue S, Nkouetoundi M, Ngollo M (1997). *Anopheles namibiensis* is anthropophilic and widespread in Cameroon. Med Vet Entomol.

[45] Menze BD, Wondji MJ, Tchapga W, Tchoupo M, Riveron JM, Wondji CS (2018). Bionomics and insecticides resistance profiling of malaria vectors at a selected site for experimental hut trials in central Cameroon. Malar J.

[46] Njan Nloga A, Robert V, Toto J, Carnevale P (1993). *Anopheles moucheti*, vecteur principal du paludisme au sud-Cameroun. Bull Liais Doc OCEAC..

[47] Nopowo F, Akono P, Tonga C, Enama L, Mbida MBIDA, Kekeunou S (2020). Écologie d’*Anopheles hancocki* Edwards, 1929 et étude de son implication dans la transmission du paludisme dans un village du bloc forestier sud-camerounais. Bull Soc Pathol Exot.

[48] The President’s Malaria Initiative (PMI)/VectorLink Project. The PMI VectorLink cameroon annual entomology report: October 2018–September 2019. Rockville, MD. The PMI VectorLink Project, Abt Associates. 2020. p. 1–74.

[49] Azari-Hamidian S, Norouzi B, Harbach RE (2019). A detailed review of the mosquitoes (Diptera: Culicidae) of Iran and their medical and veterinary importance. Acta Trop.

[50] Becker N, Petric D, Zgomba M, Boase C, Madon M, Dahl C (2010). Mosquitoes and their control.

[51] Fontenille D, Diallo M, Mondo M, Ndiaye M, Thonnon J (1997). First evidence of natural vertical transmission of yellow fever virus in *Aedes aegypti*, its epidemic vector. Trans R Soc Trop Med.

[52] Rodhain F, Perez C (1985). Precis d’entomologie médicale et vétérinaire; notions d’épidémiologie des maladies a vecteurs.

[53] Service MW, Lane RP, Crosskey RW (1993). Mosquitoes (Culicidae). Medical insects and arachnids.

[54] BUCREP. Troisième recensement générale de la population et de l’habitat. Third general population and housing census Cameroun. Yaoundé: Rapport de présentation des résultats définitifs République du Cameroun 2010. p. 65.

[55] Nkwemoh CA, Tchindjang M, Afungang RN (2017). The impact of urbanization on the vegetation of Yaounde, (Cameroon). Int J Innov Res Dev.

[56] Simard F, Nchoutpouen E, Toto JC, Fontenille D (2005). Geographic distribution and breeding site preference of *Aedes albopictus* and *Aedes aegypti* (Diptera: Culicidae) in Cameroon, Central Africa. J Med Entomol.

[57] Sighomnou D. Analyse et redéfinition des régimes climatiques et hydrologiques du Cameroun: perspectives d’évolution des ressources en eau. Thèse en Science de l'eau, Université de Yaoundé 1; 2004. p. 289.

[58] PNLP. Plan Stratégique National de Lutte contre le Paludisme au Cameroun 2019–2023. Rapport Minsante Cameroun.

[59] Fru-Cho J, Bumah VV, Safeukui I, Nkuo-Akenji T, Titanji VP, Haldar K (2014). Molecular typing reveals substantial *Plasmodium vivax* infection in asymptomatic adults in a rural area of Cameroon. Malar J.

[60] Ngassa Mbenda HG, Das A (2014). Molecular Evidence of *Plasmodium vivax* mono and mixed malaria parasite infections in duffy-negative native Cameroonians. PLoS ONE.

[61] Quakyi IA, Leke RG, Befidi-Mengue R, Tsafack M, Bomba-Nkolo D, Manga L (2000). The epidemiology of *Plasmodium falciparum* malaria in two Cameroonian villages: Simbok and Etoa. Am J Trop Med Hyg.

[62] Russo G, Faggioni G, Paganotti GM, Djeunang Dongho GB, Pomponi A, De Santis R (2017). Molecular evidence of Plasmodium vivax infection in Duffy negative symptomatic individuals from Dschang, West Cameroon. Malar J.

[63] Tchuinkam T, Nyih-Kong B, Fopa F, Simard F, Antonio-Nkondjio C, Awono-Ambene H-P (2015). Distribution of *Plasmodium falciparum* gametocytes and malaria-attributable fraction of fever episodes along an altitudinal transect in Western Cameroon. Malar J.

[64] Liu W, Li Y, Learn GH, Rudicell RS, Robertson JD, Keele BF (2010). Origin of the human malaria parasite *Plasmodium falciparum* in gorillas. Nature.

[65] Prugnolle F, Durand P, Neel C, Ollomo B, Ayala FJ, Arnathau C (2010). African great apes are natural hosts of multiple related malaria species, including *Plasmodium falciparum*. Proc Natl Acad Sci.

[66] Rayner JC, Liu W, Peeters M, Sharp PM, Hahn BH (2011). A plethora of *Plasmodium* species in wild apes: a source of human infection?. Trends Parasitol.

[67] Paupy C, Makanga B, Ollomo B, Rahola N, Durand P, Magnus J (2013). *Anopheles moucheti* and *Anopheles vinckei* are candidate vectors of ape *Plasmodium* parasites, including *Plasmodium praefalciparum* in Gabon. PLoS ONE.

[68] Sundararaman SA, Liu W, Keele BF, Learn GH, Bittinger K, Mouacha F (2013). *Plasmodium falciparum*-like parasites infecting wild apes in southern Cameroon do not represent a recurrent source of human malaria. Proc Natl Acad Sci.

[69] Atkinson CT, Dusek RJ, Woods KL, Iko WM (2000). Pathogenicity of avian malaria in experimentally-infected *hawaii amakihi*. J Wild Dis.

[70] Abella-Medrano CA, Ibáñez-Bernal S, Carbó-Ramírez P, Santiago-Alarcon D (2018). Blood-meal preferences and avian malaria detection in mosquitoes (Diptera: Culicidae) captured at different land use types within a neotropical montane cloud forest matrix. Parasitol Int.

[71] Ejiri H, Sato Y, Sawai R, Sasaki E, Matsumoto R, Ueda M (2009). Prevalence of avian malaria parasite in mosquitoes collected at a zoological garden in Japan. Parasitol Res.

[72] Okanga S, Cumming GS, Hockey PA (2013). Avian malaria prevalence and mosquito abundance in the Western Cape, South Africa. Malar J.

[73] Santiago-Alarcon D, Palinauskas V, Schaefer HM (2012). Diptera vectors of avian haemosporidian parasites: untangling parasite life cycles and their taxonomy. Biol Rev.

[74] Njabo KY, Smith TB, Yohannes E (2013). Feeding habits of culicine mosquitoes in the Cameroon lowland forests based on stable isotopes and blood meal analyses. J Parasitol Vector Biol.

[75] WHO. Global program to eliminate lymphatic filariasis: progress report. Weekly epidemiological record. 2018;93:589–604.

[76] Bakajika D, Nigo M, Lotsima J, Masikini G, Fischer, Lloyd M (2014). Filarial antigenemia and *Loa loa* night blood microfilaremia in an area without bancroftian filariasis in the Democratic Republic of Congo. Am J Trop Med Hyg.

[77] Beng AA, Esum ME, Deribe K, Njouendou AJ, Ndongmo PWC, Abong RA (2020). Mapping lymphatic filariasis in *Loa loa* endemic health districts naïve for ivermectin mass administration and situated in the forested zone of Cameroon. BMC Infect Dis.

[78] Wanji S, Esum ME, Njouendou AJ, Mbeng AA, Ndongmo PWC, Abong RA (2019). Mapping of lymphatic filariasis in loiasis areas: A new strategy shows no evidence for *Wuchereria bancrofti* endemicity in Cameroon. PLos Negl Trop Dis.

[79] Harrington LC, Edman JD, Scott TW (2001). Why Do Female *Aedes aegypti* (Diptera: Culicidae) feed preferentially and frequently on human blood?. J Med Entomol.

[80] Gonzalez JP, Josse R, Johnson ED, Merlin M, Georges AJ, Abandja J (1989). Antibody prevalence against haemorrhagic fever viruses in randomized representative central African populations. Res Virol.

[81] Tchuandom SB, Tchouangueu TF, Antonio-Nkondjio C, Lissom A, Djang JON, Atabonkeng EP (2018). Seroprevalence of dengue virus among children presenting with febrile illness in some public health facilities in Cameroon. Pan Afr Med J.

[82] Tchuandom SB, Lissom A, Ateba GHM, Tchouangueu TF, Tchakounte C, Ayuk AR (2020). Dengue virus serological markers among potential blood donors: an evidence of asymptomatic dengue virus transmission in Cameroon. Pan Afr Med J.

[83] Auguste AJ, Kaelber JT, Fokam EB, Guzman H, Carrington CVF, Erasmus JH (2015). A Newly isolated reovirus has the simplest genomic and structural organization of any reovirus. J Virol.

[84] Brottes H, Rickenbach A, Bres P, Salaun J-J, Ferrara L (1966). Les arbovirus au Cameroun, isolement à partir des moustiques. Bull World Health Org.

[85] Brottes H, Rickenbach A, Bres P, Williams MC, Salaun JJ, Ferrara L (1969). Le virus Okola (YM 50/64) nouveau prototype d’arbovirus isolé au Cameroun à partir de moustiques. Ann Inst Pasteur.

[86] Fokam EB, Levai LD, Guzman H, Amelia PA, Titanji VPK, Tesh RB (2010). Silent circulation of arboviruses in Cameroon. East Afr Med J.

[87] Kuniholm MH, Wolfe ND, Huang CYH, Mpoudi-Ngole E, Tamoufe U, Burke DS (2006). Seroprevalence and distribution of Flaviviridae, Togaviridae, and Bunyaviridae arboviral infections in rural Cameroonian adults. Am J Trop Med Hyg.

[88] Macnamara FN (1953). The Susceptibility of chicks to Semliki Forest Virus (Kumba Strain). Ann Trop Med Parasitol.

[89] Poirier A, Germain M, Rickenbach A, Eouzan J-P (1969). Recherches sur le réservoir animal d’arbovirus dans une région forestière du Cameroun: communication préliminaire. Bull Soc Pathol Exot.

[90] Rickenbach A, Germain M, Eouzan J-P, Poirier A (1969). Recherches sur l’epidemiologie des arboviroses dans une region forestiere du Sud-Cameroun. Bull Soc Pathol Exot.

[91] Zeller HG, Bessin R, Thiongane Y, Bapetel I, Teou K, Ala MG (1995). Rift Valley fever antibody prevalence in domestic ungulates in Cameroon and several West African countries (1989–1992) following the 1987 Mauritanian outbreak. Res Virol.

[92] Poueme R, Stoek F, Nloga N, Awah-Ndukum J, Rissmann M, Schulz A (2019). Seroprevalence and associated risk factors of rift valley fever in domestic small ruminants in the North Region of Cameroon. Vet Med Int.

[93] Tchuandom SB, Tchadji JC, Tchouangueu TF, Biloa MZ, Atabonkeng EP, Fumba MIM (2019). A cross-sectional study of acute dengue infection in paediatric clinics in Cameroon. BMC Pub Health.

[94] Peyrefitte CN, Rousset D, Pastorino BA, Pouillot R, Bessaud M, Tock F (2007). Chikungunya virus, Cameroon, 2006. Emerg Infect Dis.

[95] Yousseu FBS, Nemg FBS, Ngouanet SA, Mekanda FMO, Demanou M (2018). Detection and serotyping of dengue viruses in febrile patients consulting at the New-Bell District Hospital in Douala, Cameroon. PLoS ONE.

[96] Gake B, Vernet MA, Leparc-Goffart I, Drexler JF, Gould EA, Gallian P (2017). Low seroprevalence of Zika virus in Cameroonian blood donors. Braz J Infect Dis.

[97] Nemg F, Sado F, Evouna Mbarga A, Bigna JJ, Melong A, Ntoude A (2019). Investigation of an Outbreak of Dengue virus serotype 1 in a rural area of Kribi, South Cameroon: a cross-sectional study. Intervirol.

[98] Demanou M, Antonio-Nkondjio C, Ngapana E, Rousset D, Paupy C, Manuguerra J-C (2010). Chikungunya outbreak in a rural area of Western Cameroon in 2006: a retrospective serological and entomological survey. BMC Res Notes.

[99] Demanou M, Pouillot R, Grandadam M, Boisier P, Kamgang B, Hervé JP (2014). Evidence of dengue virus transmission and factors associated with the presence of anti-dengue virus antibodies in humans in three major towns in Cameroon. PLoS Negl Trop Dis.

[100] Krippner R, von Laer G (2002). First confirmed dengue-1 fever cases reported from Cameroon. J Travel Med.

[101] Ayala D, Costantini C, Ose K, Kamdem GC, Antonio-Nkondjio C, Agbor J-P (2009). Habitat suitability and ecological niche profile of major malaria vectors in Cameroon. Malar J.

[102] Bamou R, Mbakop LR, Kopya E, Ndo C, Awono-Ambene P, Tchuinkam T (2018). Changes in malaria vector bionomics and transmission patterns in the equatorial forest region of Cameroon between 2000 and 2017. Parasit Vectors.

[103] Mayi MPA, Bamou R, Djiappi-Tchamen B, Fontaine A, Jeffries CL, Walker T (2020). Habitat and seasonality affect mosquito community composition in the West Region of Cameroon. Insects.

[104] Mayi MPA, Foncha DF, Kowo C, Tchuinkam T, Brisco K, Anong DN (2019). Impact of deforestation on the abundance, diversity, and richness of *Culex* mosquitoes in a southwest Cameroon tropical rainforest. J Vector Ecol.

[105] Mayi MPA, Bamou R, Djapi-Tchamen B, Carelle D-T, Fontaine A, Antonio-Nkondjio C (2019). A mosquito survey along a transect of urbanization in Dschang, West Region of Cameroon, reveals potential risk of arbovirus spillovers. Ecology.

[106] Pajot F-X, Ségers LG (1964). Notes sur la biologie d’Anopheles hargreavesi (Evans) 1927, et d’Anopheles paludis Theobald 1900, dans le Sud de la zone d’entrainement de Yaoundé (Cameroun), le long du fleuve Nyong. Cahiers ORSTOM Entomol Méd Parasitol..

[107] Rageau J, Adam J-P (1953). Culicinæ du Cameroun. Ann Parasitol Hum Comp.

[108] Rageau J, Adam J-P (1953). Note complémentaire sur les Culicinæ du Cameroun. Ann Parasitol Hum Comp.

[109] Salaun JJ, Rickenbach A, Brès P, Brottes H, Germain M, Eouzan J-P (1969). Les arbovirus isolés à partir de moustiques au Cameroun. Bull World Health Org.

[110] Takougang I, Same Ekobo A, Ebo’o Eyenga V, Enyong P (1994). Vectorial fauna at the site of the future dam at Memve’ele(Cameroon). Bull Soc Path Exot.

[111] Zumpt F (1937). Culicid Studies in the Plantation Region of the Cameroon Mountain. Tropenpflanzer..

[112] Coetzee M (2020). Key to the females of Afrotropical *Anopheles* mosquitoes (Diptera: Culicidae). Malar J.

[113] Elanga-Ndille E, Nouage L, Binyang A, Assatse T, Tene-Fossog B, Tchouakui M (2019). Overexpression of Two members of d7 salivary genes family is associated with pyrethroid resistance in the malaria vector *Anopheles funestus* s.s. but not in *Anopheles gambiae* in Cameroon. Genes.

[114] Irish SR, Kyalo D, Robert W, Snow, Coetzee M (2020). Updated list of *Anopheles* species (Diptera: Culicidae) by country in the Afrotropical Region and associated islands. Zootaxa.

[115] Kyalo D, Amratia P, Mundia CW, Mbogo CM, Coetzee M, Snow RW (2017). A geo-coded inventory of anophelines in the Afrotropical Region south of the Sahara: 1898–2016. Wellcome Open Res.

[116] Mouchet J, Gariou J, Hamon J (1960). Note faunistique sur les Moustiques des montagnes de l’ouest-Cameroun Presence de neuf formes de Culicidae nouvelles pour le Cameroun. Bull l’IFANT SXII.

[117] Djamouko-Djonkam L, Nkahe DL, Kopya E, Talipouo A, Ngadjeu CS, Doumbe-Belisse P (2020). Implication of *Anopheles funestus* in malaria transmission in the city of Yaoundé, Cameroon. Parasite.

[118] Doumbe-Belisse P, Ngadjeu CS, Sonhafouo-Chiana N, Talipouo A, Djamouko-Djonkam L, Kopya E (2018). High malaria transmission sustained by *Anopheles gambiae* s.l. occurring both indoors and outdoors in the city of Yaoundé, Cameroon. Wellcome Open Res.

[119] Ndo C, Poumachu Y, Metitsi D, Awono-Ambene HP, Tchuinkam T, Gilles JLR (2018). Isolation and characterization of a temperature-sensitive lethal strain of *Anopheles arabiensis* for SIT-based application. Parasit Vectors.

[120] Nwane P, Etang J, Chouaibou M, Toto JC, Kerah-Hinzoumbé C, Mimpfoundi R (2009). Trends in DDT and pyrethroid resistance in *Anopheles gambiae* s.s. populations from urban and agro-industrial settings in southern Cameroon. BMC Infect Dis.

[121] Talipouo A, Ngadjeu CS, Doumbe-Belisse P, Djamouko-Djonkam L, Sonhafouo-Chiana N, Kopya E (2019). Malaria prevention in the city of Yaoundé: knowledge and practices of urban dwellers. Malar J.

[122] Bamou R, Kopya E, Djamouko-Djonkam L, Awono-Ambene P, Tchuinkam T, Njiokou F (2020). Assessment of the Anophelinae blood seeking bionomic and pyrethroids resistance of local malaria vectors in the forest region of Southern Cameroon. J Entomol Zool Studies.

[123] Talom AD, Essoung MA, Gbankoto A, Tchigossou G, Akoton R, Sahabi BBA (2020). A preliminary analysis on the effect of copper on *Anopheles coluzzii* insecticide resistance in vegetable farms in Benin. Sci Rep.

[124] Bamou R, Sonhafouo-Chiana N, Mavridis K, Tchuinkam T, Wondji CS, Vontas J (2019). Status of insecticide resistance and its mechanisms in *Anopheles gambiae* and *Anopheles coluzzii* populations from forest settings in South Cameroon. Genes.

[125] Menze BD, Kouamo MF, Wondji MJ, Tchapga W, Tchoupo M, Kusimo MO (2020). An Experimental hut evaluation of PBO-based and pyrethroid-only nets against the malaria vector *Anopheles funestus* reveals a loss of bed nets efficacy associated with GSTE2 metabolic resistance. Genes.

[126] Tchouakui M, Riveron Miranda J, Mugenzi LMJ, Djonabaye D, Wondji MJ, Tchoupo M (2020). Cytochrome P450 metabolic resistance (CYP6P9a) to pyrethroids imposes a fitness cost in the major African malaria vector *Anopheles funestus*. Heredity.

[127] Harbach RE, Howard TM (2007). Index of currently recognized mosquito species (Diptera: Culicidae). Bull J Euro Mosq Contr Assoc.

[128] Akono PN, Mbida AM, Ambene PA (2018). Habitats larvaires et sensibilité des vecteurs du paludisme aux insecticides dans des localités (semi-urbaine et rurale) de la région du littoral Camerounais: données préliminaires. Rev Ecol.

[129] Foko D, Zebaze J, Ajeagah G, Takougang I, Tamesse L (2016). Diversité culicidienne dans un cours d’eau anthropisé de la ville de Yaoundé, Cameroun : importance des facteurs environnementaux. Afr Sci.

[130] Nchoutpouen E, Talipouo A, Djiappi-Tchamen B, Djamouko-Djonkam L, Kopya E, Ngadjeu CS (2019). *Culex* species diversity, susceptibility to insecticides and role as potential vector of *Lymphatic filariasis* in the city of Yaoundé, Cameroon. PLoS Negl Trop Dis.

[131] Fon TE, Ukaga CN, Yongabi KA, Nwoke BEB (2017). Spatial distribution and habitat characterization of mosquito larvae in Bamenda, Cameroon. Ind J Med Res Pharm Sci.

[132] Hopkins GHE (1952). Mosquitoes of the Ethiopian region I Larval bionomics of mosquitoes and taxonomy of Culicine Larvae.

[133] Vinogradova E, Shaikevich E, Ivanitsky A (2007). A study of the distribution of the *Culex pipiens* complex (Insecta: Diptera: Culicidae) mosquitoes in the European part of Russia by molecular methods of identification. Compar Cytogen.

[134] Gomes B, Sousa CA, Vicente JL, Pinho L, Calderón I, Arez E (2013). Feeding patterns of molestus and pipiens forms of *Culex pipiens* (Diptera: Culicidae) in a region of high hybridization. Parasit Vectors.

[135] Kumar K, Katyal R, Gill KS (2002). Feeding pattern of anopheline and culicine mosquitoes in relation to biotopes and seasons in Delhi and environs. J Commun Dis.

[136] Amraoui F, Krida G, Bouattour A, Rhim A, Daaboub J, Harrat Z (2012). *Culex pipiens*, an experimental efficient vector of West Nile and Rift Valley vever Viruses in the Maghreb Region. PLoS ONE.

[137] Brustolin M, Talavera S, Núñez AI, Santamaria C, Rivas R, Pujol N (2017). Rift Valley fever virus and European mosquitoes: vector competence of *Culex pipiens* and *Stegomyia albopicta* (*Aedes albopictus*): European mosquitoes as vectors of RVFV. Med Vet Entomol.

[138] Dohm DJ, O’Guinn ML, Turell MJ (2002). Effect of environmental temperature on the ability of *Culex pipiens* (Diptera: Culicidae) to transmit West Nile virus. J Med Entomol.

[139] Vloet RPM, Vogels CBF, Koenraadt CJM, Pijlman GP, Eiden M, Gonzales JL (2017). Transmission of Rift Valley fever virus from European-breed lambs to *Culex pipiens* mosquitoes. PLoS Negl Trop Dis.

[140] Farid HA, Hammad RE, Hassan MM, Morsy ZS, Kamal IH, Weil GJ (2001). Detection of *Wuchereria bancrofti* in mosquitoes by the polymerase chain reaction: a potentially useful tool for large-scale control programmes. Trans R Soc Trop Med Hyg.

[141] Nasution S, Adhiyanto C, Indahwati E (2018). Preliminary study of *Wuchereria bancrofti* L3 larvae detection in *Culex quinquefasciatus* as vector potential of filariasis inendemic area of south Tangerang, by utilizing PCR assay for L3-activated cuticlin transcript mRNA gene and TPH-1 gene. Ind J Trop Infect Dis.

[142] Simonsen PE, Mwakitalu ME (2013). Urban lymphatic filariasis. Parasitol Res.

[143] Hougard J, Mbentengam R, Lochouarn L, Escaffre H, Darriet F, Barbazan P (1993). Campaign against *Culex quinquefasciatus* using *Bacillus sphaericus*: results of a pilot project in a large urban area of equatorial Africa. Bull World Health Org.

[144] Barbazan P, Baldet T, Darriet F, Escaffre H, Djoda DH, Hougard J-M (1997). Control of *Culex quinquefasciatus* (Diptera: Culicidae) with *Bacillus sphaericus* in Maroua, Cameroon. J Am Mosq Control Assoc.

[145] Griffin L, Knight J (2012). A review of the role of fish as biological control agents of disease vector mosquitoes in mangrove forests: reducing human health risks while reducing environmental risk. Wetlands Ecol Manage.

[146] Kweka EJ, Baraka V, Mathias L, Mwangonde B, Baraka G, Lyaruu L (2018). Ecology of *Aedes* mosquitoes, the major vectors of arboviruses in human population. Dengue Fever-A resilient threat in the face of innovation.

[147] Gaffigan TV., Wilkerson RC, Pecor JE, Stoffer JA, Anderson T. Systematic catalog of Culicidae. 2015. https://wrbu.si.edu/vectorspecies. Accessed 25 Apr 2020.

[148] Tedjou AN, Kamgang B, Yougang AP, Njiokou F, Wondji CS (2019). Update on the geographical distribution and prevalence of *Aedes aegypti* and *Aedes albopictus* (Diptera: Culicidae), two major arbovirus vectors in Cameroon. PLoS Negl Trop Dis.

[149] Fontenille D, Toto JC (2001). *Aedes* (Stegomyia) *albopictus* (Skuse), a potential new Dengue vector in southern Cameroon. Emerg Infect Dis.

[150] Kamgang B, Yougang AP, Tchoupo M, Riveron JM, Wondji C (2017). Temporal distribution and insecticide resistance profile of two major arbovirus vectors *Aedes aegypti* and *Aedes albopictus* in Yaoundé, the capital city of Cameroon. Parasit vectors.

[151] Ngo H, Akono P, Ngo V, Nko’o E, Tonga C. Adaptation compétitive d’Aedes albopictus Skuse,  (1894). en présence d’*Aedes aegypti* Linné, 1862 dans quelques gîtes larvaires temporaires de la ville de Douala (Cameroun) dans un contexte de résistance aux pyréthrinoïdes. Bull Soc Path Exot.

[152] Sota T, Mogi M (1992). Survival time and resistance to desiccation of diapause and non-diapause eggs of temperate *Aedes* (Stegomyia) mosquitoes. Entomol Exp Appl.

[153] Akıner MM, Öztürk M, Başer AB, Günay F, Hacıoğlu S, Brinkmann A (2019). Arboviral screening of invasive *Aedes* species in northeastern Turkey: West Nile virus circulation and detection of insect-only viruses. PLoS Negl Trop Dis.

[154] Chouin-Carneiro T, Vega-Rua A, Vazeille M, Yebakima A, Girod R, Goindin D (2016). Differential susceptibilities of *Aedes aegypti* and *Aedes albopictus* from the Americas to Zika virus. PLoS Negl Trop Dis.

[155] Gratz NG (2004). Critical review of the vector status of *Aedes albopictus*. Med Vet Entomol.

[156] Gubler DJ (2002). The global emergence/resurgence of arboviral diseases as public health problems. Arch Med Res.

[157] Palatini U, Miesen P, Carballar-Lejarazu R, Ometto L, Rizzo E, Tu Z (2017). Comparative genomics shows that viral integrations are abundant and express piRNAs in the arboviral vectors *Aedes aegypti* and *Aedes albopictus*. BMC Genomics.

[158] Kamgang B, Vazeille M, Tedjou AN, Wilson-Bahun TA, Yougang AP, Mousson L (2019). Risk of dengue in Central Africa: vector competence studies with *Aedes aegypti* and *Aedes albopictus* (Diptera: Culicidae) populations and dengue 2 virus. PLoS Negl Trop Dis.

[159] Kamgang B, Vazeille M, Yougang AP, Tedjou AN, Wilson-Bahun TA, Mousson L (2019). Potential of *Aedes albopictus* and *Aedes aegypti* (Diptera: Culicidae) to transmit yellow fever virus in urban areas in Central Africa. Emerg Microbes Infect.

[160] Service MW (1990). Handbook to the Afrotropical toxorhynchitine and culicine mosquitoes, excepting *Aedes* and *Culex*.

[161] Edwards FW. Mosquitoes of the Ethiopian Region. HI.—Culicine Adults and Pupae. Mosquitoes of the Ethiopian Region HI—Culicine adults and pupae order of the Trustees of the British Museum. 1941. p. 1–499.

[162] Lounibos LP (1978). Mosquito breeding and opposition stimulant in fruit husks. Ecol Entomol.

[163] Haddow AJ (1946). The mosquitoes of Bwamba County, Uganda: IV.—studies on the genus eretmapodites theobald. Bull Entomol Res.

[164] Bauer JH (1928). The transmission of yellow fever by mosquitoes other than *Aedes Aegypti*. Am J Trop Med Hyg.

[165] Smithburn KC, Haddow AJ, Gillett JD (1948). Rift Valley Fever. Isolation of the virus from wild mosquitoes. Br J Exp Pathol.

[166] Worth CB, Paterson HE, de Meillon B (1961). The incidence of arthropod-borne viruses in a population of culicine mosquitoes in Tongaland, Union of South Africa. Am J Trop Med Hyg.

[167] Salaun JJ, Rickenbach A, Bres P, Brottes H, Germain M, Eouzan J-P (1969). Le virus Nkolbisson (YM 31/65) nouveau prototype d’arbovirus isolé au Cameroun. Ann Inst Pasteur.

[168] Ardoin P, Simpson D (1965). Antigenic relationships between the Nyando virus and 2 viruses isolated in Ethiopia from collections of Eretmapodites. Bull Soc Pathol Exot.

[169] Gilotra SK, Shah KV (1967). Laboratory studies on transmission of chikungunya virus by mosquitoes. Am J Epidemiol.

[170] Hartberg WK, Gerberg EJ (1971). Laboratory colonization of *Aedes simpsoni* (Theobald) and *Eretmapodites quinquevittatus* Theobald. Bull World Health Organ.

[171] Rickenbach A, Gonidec L (1976). L’incidence des arbovirus isolés des moustiques dans une région forestière du Sur Cameroun, la région de Yaoundé. Bull Soc Pathol Exot.

[172] Foster WA, Walker ED (2019). Mosquitoes (Culicidae). Med Vet Entomol.

[173] Sardelis MR, Turell MJ, Dohm DJ, O’Guinn ML (2001). Vector competence of selected North American *Culex* and *Coquillettidia* mosquitoes for West Nile virus. Emerg Infect Dis.

[174] White GB, Faust C (2014). Medical acarology and entomology. Manson’s tropical infectious diseases.

[175] Kenny E, Ruber E (1992). Effectiveness of aerially applied Arosurf MSF in the control of the cattail mosquito, *Coquillettidia perturbans*. J Am Mosq Control Assoc.

[176] Sinegre G, Cousserans J, Vico G, Crespo O (1971). Susceptibility of Mansonia (*C*.) *richiardii* larvae to some insecticides. The effect of Abate on larvae and wildlife. Cahiers ORSTOM.

[177] Henderson JP, Westwood R, Galloway T (2006). An assessment of the effectiveness of the mosquito magnet pro model for suppression of nuisance mosquitoes. J Am Mosq Control Assoc.

[178] Kaufman PE, Butler JF, Nelson C (2008). Evaluation of the mosquito sentinel 360 trap in florida residential environments. J Am Mosq Control Assoc.

[179] Sérandour J, Rey D, Raveton M (2006). Behavioural adaptation of *Coquillettidia (Coquillettidia) richiardii* larvae to underwater life: environmental cues governing plant–insect interaction. Entomol Exp Appl.

[180] Sérandour J, Willison J, Thuiller W, Ravanel P, Lempérière G, Raveton M (2010). Environmental drivers for *Coquillettidia* mosquito habitat selection: a method to highlight key field factors. Hydrobiologia.

[181] Sjogren S, Batzer D, Juenemann M (1986). Evaluation of Methoprene, temephos and *Bacillus thuringiensis* var. israelensis against *Coquillettidia perturbans* larvae in Minnosota. J Am Mosq Contol Assoc.

[182] Biswas D, Ghosh A, Chowdhury N, Chandra G (2011). Man biting activity of *Mansonia annulifera* and *Mansonia indiana* in Burdwan, West Bengal. In J Entomol Res.

[183] Onapa AW, Pedersen EM, Reimert CM, Simonsen PE (2007). A role for *Mansonia uniformis* mosquitoes in the transmission of lymphatic filariasis in Uganda?. Acta Trop.

[184] Smith A (1955). On the transmission of bancroftial filariasis on Ukara Island, Tanganyika. IV.—host-preferences of mosquitos and the incrimination of *Anopheles gambiae* Giles and *An. funestus* Giles as vectors of bancroftial filariasis. Bull Entomol Res.

[185] Apiwathnasorn C, Samung Y, Prummongkol S, Asavanich A, Komalamisra N (2006). Surveys for natural host plants of *Mansonia* mosquitoes inhabiting Toh Daeng peat swamp forest, Narathiwat Province, Thailand, Southeast Asian. J Trop Med Pub Health.

[186] McNeel TE (1931). A Method for locating the larvae of the mosquito. Mansonia Sci.

[187] Dijk W (1958). Transmission of *Wuchereria bancrofti* in Netherlands NewGuinea. Trop GeogrMed.

[188] Ughasi J, Bekard H, Coulibaly M, Adabie-Gomez D, Gyapong J, Appawu M (2012). *Mansonia africana* and *Mansonia uniformis* are vectors in the transmission of *Wuchereria bancrofti* lymphatic filariasis in Ghana. Parasit Vectors.

[189] Wharton RH (1957). Studies on filariasis in Malaya: the efficiency of *Mansonia longipalpis* as an experimental vector of *Wuchereria malayi*. Ann Trop Med Parasitol.

[190] Dietrich CF, Chaubal N, Hoerauf A, Kling K, Piontek MS, Steffgen L (2019). Review of dancing parasites in lymphatic filariasis. Ultrasound Int Open.

[191] Donald C, Siriyasatien, Kohl A (2020). *Toxorhynchites* species: a review of current knowledge. Insect.

[192] Conradie R, Cook C, du Preez L, Jordaan A, Netherlands E (2017). Ultrastructural comparison of *Hepatozoon ixoxo* and *Hepatozoon theileri* (Adeleorina: Hepatozoidae), parasitising South African Anurans. J Eukaryot Microbiol.

[193] Netherlands E, Cook C, du Preez L, Vanhove M, Brendonck L, Smit N (2019). An overview of the Dactylosomatidae (Apicomplexa: Adeleorina: Dactylosomatidae), with the description of *Dactylosoma kermiti* n. sp. parasitising *Ptychadena anchietae* and *Sclerophrys gutturalis* from South Africa. Int J Parasitol Parasites Wildl.

[194] Netherlands E, Svitin R, Cook C, Smit N, Brendonck L, Vanhove M (2020). *Neofoleyellides boerewors* n. gen. n. sp. (Nematoda: Onchocercidae) parasitising common toads and mosquito vectors: morphology, life history, experimental transmission and host-vector interaction in situ. Int J Parasitol.

[195] Edwards (1914). New species of Culicidae in the British Museum, with notes on the genitalia of some African *Culex*. Bull Ent Res.

[196] Nebbak A, Koumare S, Willcox AC, Berenger J-M, Raoult D, Almeras L (2018). Field application of MALDI-TOF MS on mosquito larvae identification. Parasitology.

[197] Niare S, Tandina F, Davoust B, Doumbo O, Raoult D, Parola P (2018). Accurate identification of *Anopheles gambiae* Giles trophic preferences by MALDI-TOF MS. Infect Gen Evol.

[198] Raharimalala FN, Andrianinarivomanana TM, Rakotondrasoa A, Collard JM, Boyer S (2017). Usefulness and accuracy of MALDI-TOF mass spectrometry as a supplementary tool to identify mosquito vector species and to invest in development of international database. Med Vet Entomol.

[199] Tandina F, Laroche M, Davoust B, Doumbo OK, Parola P (2018). Blood meal identification in the cryptic species *Anopheles gambiae* and *Anopheles coluzzii* using MALDI-TOF MS. Parasite.

[200] Boucheikhchoukh M, Laroche M, Aouadi A, Dib L, Benakhla A, Raoult D (2018). MALDI-TOF MS identification of ticks of domestic and wild animals in Algeria and molecular detection of associated microorganisms. Comp Immunol Microbiol Infect Dis.

[201] Cohuet A, Simard F,  Toto J, Kengne P, Coetzee M (2003). Species identification within the *Anopheles funestus* group of malaria vectors in Cameroon and evidence for a new species. Am J Trop Med Hyg.

