# Supplementary material for: An update on the mosquito fauna and mosquito-borne diseases distribution in Cameroon
Source: Parasit Vectors. 2021 Oct 11;14:527. doi: 10.1186/s13071-021-04950-9 (PMC8507310; doi:10.1186/s13071-021-04950-9)
Supplement: Supplementary file 5 — Additional file 5: Table S1. Anopheles species composition, pathogens transmitted and control interventions in Cameroon. Table S2. Culex species composition, pathogens transmitted and control interventions in Cameroon. Table S3. Aedes species composition, pathogens transmitted and control interventions in Cameroon. Table S4. Eretmapodites species composition, pathogens transmitted and control interventions in Cameroon. Table S5. Coquillettidia species composition in Cameroon, pathogens transmitted and control interventions. Table S6. Mansonia species composition, pathogens transmitted and control interventions in Cameroon. Table S7. Mosquito species of unknown or minimal medical and veterinary importance: species composition, pathogens transmitted and control interventions in Cameroon. [file 13071_2021_4950_MOESM5_ESM.docx]

**Additional file 5: Table S1.** *Anopheles* mosquitoes (60 valid species plus a putative new species not formally described), pathogens transmitted and control interventions in Cameroon. Species are grouped by subgenera.

| **Species names** | **Pathogens** | **Main vector species** | **Vector control measures** |
| --- | --- | --- | --- |
| ***Christya***  *An. implexus*, *An. okuensis* | *P. vivax*, *P. falciparum*, *P. malariae*, *P. ovale* | *An. gambiae*, *An. arabiensis*, *An. coluzzii*, *An. nili*, *An. funestus*, *An. moucheti* | LLINs, IRS, LSM, coils, repellents, good house design |
| ***Anopheles***  *An. ziemanni*, *An. coustani*, *An. obscurus*, *An. paludis*, *An. namibiensis*, *An. concolor*, *An. tenebrosus* |  |  |  |
| ***Cellia***  *An. arabiensis*, *An. moucheti*, *An. nili*, *An. natalensis*, *An.* *multicinctus*, *An. squamosus*, *An. cydippis*, *An. ardensis*, *An. brohieri*, *An. bervoetsi*, *An. brunnipes*, *An. wellcomei*, *An. melas*, *An. buxtoni*, *An. freetownensis*, *An. carnevalei*, *An. christyi*, *An. jebudensis*, *An. coluzzii*, *An. gambiae*, *An. hancocki, An. marshallii*, *An. maculipalpis*, *An. deemingi*, *An. longipalpis*, *An. leesoni*, *An. demeilloni*, *An. domicolus*, *An. hervyi*, *An. dualaensis*, *An. hargreavesi*, *An. eouzani*, *An. funestus*, *An. smithii*, *An. flavicosta*, *An. cinctus*, *An. pharoensis*, *An. pretoriensis*, *An. rageaui*, *An. rhodesiensis*, *An. ovengensis*, *An. rivolurum*-like ^a^, *An. somalicus*, *An. rivulorum*, *An. squamosus*, *An. rufipes*, *An. sergentii*, *An. theileri*, *An. njombiensis*, *An. dureni*, *An. parensis*, *An. mousinshoi* |  |  |  |

Abbreviations: LLINs: long lasting insecticide treated nets; IRS: indoor residual spraying; LSM: larval source management.

^a^ Species not yet formally named, originally reported by Cohuet et al. [201]. This species is present in the list of Irish et al. [114].

**Additional file 5: Table S2.** *Culex* mosquitoes (67 species and two subspecies), pathogens transmitted and control interventions in Cameroon. Species are grouped by subgenera.

| **Species** | **Pathogens** | **Main vector species** | **Vector control measures** |
| --- | --- | --- | --- |
| ***Culex***  *Cx. antennatus*, *Cx. argenteopunctatus*, *Cx. chorleyi*, *Cx. decens*, *Cx. duttoni*, *Cx. gameti*, *Cx. pruina*, *Cx. neavei*, *Cx. grahamii*, *Cx. guiarti*, *Cx. ingrai*, *Cx.* *invidiosus*, *Cx. pajoti*, *Cx. philipi*, *Cx.* *ornatothoracis*, *Cx. perfidiosus*, *Cx.* *perfuscus*, *Cx. pipiens* s.l., *Cx. weschei*, *Cx. quasiguiarti*, *Cx. quinquefasciatus*, *Cx. schwetzi*, *Cx. sitiens*, *Cx. telesilla*, *Cx. thalassius*, *Cx. toroensis*, *Cx. trifoliatus*, *Cx. trifilatus*, *Cx. watti*, *Cx. tritaeniorhynchus*, *Cx. univitattus*, *Cx. vansomeni* | Ntaya Virus, *Plasmodium* spp. (Avian malaria parasites), Middelburg virus, Spondweni virus, Nkolbisson virus, Sindbis virus, Usutu virus, Wesselbron virus, *Wuchereria bancrofti*, Rift Valley fever virus | *Cx. quinquefasciatus*, *Cx. nebulosus*, *Cx. neavei*, *Cx. telesilla*, *Cx*. *annulioris*, *Cx. poicilipes* | LLINs, LSM, IRS, repellents, house design |
| ***Culiciomyia***  *Cx. apicopilosus*, *Cx. cinerellus*, *Cx. cinereus*, *Cx. eouzani*, *Cx. grenieri*, *Cx. harleyi*, *Cx. lanzaroi*, *Cx. macfiei*, *Cx. nebulosus*, *Cx. pseudosubequalis*, *Cx. semibrunneus*, *Cx. subaequalis* |  |  |  |
| ***Eumelanomyia***  *Cx. adami*, *Cx. albiventris*, *Cx. rima*, *Cx. andreanus*, *Cx. garioui*, *Cx. helenae*, *Cx.* *fimbriforceps*, *Cx. horridus*, *Cx. mijanae*, *Cx. insignis*, *Cx. wansoni*, *Cx. inconspicuosus*, *Cx. kingianus*, *Cx. laplantei*, *Cx. sunyaniensis*, *Cx. pseudoandreanus*, *Cx. subrima*, *Cx*. *rubinotus*, *Cx. wigglesworthi*, *Cx. simpliciforceps* |  |  |  |
| ***Kitzmilleria***  *Cx. moucheti* |  |  |  |
| ***Oculeomyia***  *Cx. annulioris* *annulioris*, *Cx. annulioris consimilis*, *Cx. bitaeniorhynchus*, *Cx. poicilipes* |  |  |  |

Abbreviations: LLINs: long lasting insecticide treated nets; IRS: indoor residual spraying; LSM: larval source management.

**Additional file 5: Table S3.** *Aedes* mosquitoes (77 species and one subspecies) composition, pathogens transmitted and control interventions in Cameroon. Species are grouped by subgenera.

| **Species** | **Pathogens** | **Main vector species** | **Vector control measures** |
| --- | --- | --- | --- |
| ***Aedimorphus***  *Ae. cumminsii*, *Ae. domesticus*, *Ae. ebogoensis*, *Ae. fowleri*, *Ae. leptolabis*, *Ae. mattinglyi*, *Ae. ochraceus*, *Ae. pubescens*, *Ae. quasiunivittatus*, *Ae. rickenbachi*, *Ae. tauffliebi*, *Ae. vexans*, *Ae. abnormalis*, *Ae. gibbinsi*, *Ae. africanus*, *Ae. wigglesworthi* | MIDV, WESV, Nkolbisson virus, Bunyamwera virus group, Eretmapodites 147 virus, Fako virus, Rift Valley fever virus, Dengue virus, Chikungunya; Zika virus, Yellow fever virus | *Ae. aegypti*, *Ae. albopictus*, *Ae. luteocaphalus*, *Ae. simpsoni*, *Ae. tarsalis* | LSM, aerial spray with DDT, repellents |
| ***Albuginosus***  *Ae. capensis*, *Ae. marshallii*, *Ae. haworthi*, *Ae. stokesi* |  |  |  |
| ***Bifidistylus***  *Ae. boneti*, *Ae. lamborni* |  |  |  |
| ***Catageiomyia***  ***Ae. argenteopunctatus***, *Ae. falabreguesi*, *Ae. filicis*, *Ae. grenieri*, *Ae. hopkinsi*, *Ae. insolens*, *Ae. irritans*, *Ae. mutila*, *Ae. nyounae*, *Ae. phyllolabis*, *Ae. pseudotarsalis*, ***Ae. minutus***, *Ae. tarsalis*, *Ae. yangambiensis*, *Ae. yvonneae* |  |  |  |
| ***Diceromyia***  *Ae. mefouensis*, *Ae. furcifer* |  |  |  |
| ***Elpeytonius***  *Ae. simulans* |  |  |  |
| ***Fredwardsius***  *Ae. vittatus* |  |  |  |
| ***Mucidus***  *Ae. grahamii*, *Ae. mucidus* |  |  |  |
| ***Neomelanoconion***  *Ae. carteri*, *Ae. circumluteolum*, *Ae. mcintoshi*, *Ae. monotrichus*, *Ae. jamoti*, ***Ae. palpalis***, ***Ae. pogonurus***, ***Ae. taeniarostris*** |  |  |  |
| ***Polyleptiomyia***  ***Ae. albocephalus*** |  |  |  |
| ***Pseudarmigeres***  *Ae. argenteoventralis dunni,* *Ae. kummi* |  |  |  |
| ***Stegomyia***  *Ae. aegypti*, ***Ae. albopictus***, ***Ae.*** ***apicoargenteus***, *Ae. blacklocki*, *Ae. bromeliae*, ***Ae. calceatus***, *Ae. corneti*, *Ae. denderensis*, ***Ae. dendrophilus***, *Ae. fraseri*, *Ae. hansfordi*, *Ae. maxgermaini*, ***Ae.*** ***luteocephalus***, ***Ae. metallicus***, ***Ae. pseudoafricanus***, *Ae. schwetzi*, *Ae. simpsoni*, ***Ae. soleatus*** |  |  |  |
| ***Zavortinkius***  *Ae. geoffroyi*, *Ae. huangae*, *Ae. longipalpis* |  |  |  |
| ***Hopkinsius***  *Ae. wellmanii*, *Ae. ingrami* |  |  |  |
| ***Pseudalbuginosus***  *Ae. grjebinei* |  |  |  |

Abbreviations: MIDV: Middleburg virus; WESV: Wesselsbron virus; DDT: Dichlorodiphenyltrichloroethane. Species in bold are placed in a different genus in the list published by Habarch [2].

Species in boldface are written according to the “Valid Species (composite *Aedes*)”, but have different names in the “Valid Species List” [2]. Both lists are available at the Mosquito Taxonomy Inventory homepage [2].

**Additional file 5: Table S4.** *Eretmapodites* mosquitoes (31 species and one subspecies) composition, pathogens transmitted and control interventions in Cameroon. Species are grouped by species groups.

| **Species** | **Pathogens** | **Main vector species** | **Vector control measures** |
| --- | --- | --- | --- |
| **Chrysogaster group**  *Er. brottesi*, *Er. chrysogaster*, *Er. gilletti*, *Er. grahami*, *Er. haddowi*, *Er. harperi*, *Er. intermedius*, *Er. pauliani*, *Er. semisimplicipes* | NTAV, Semliki forest virus, Usutu virus, MIDV, SPOV, NKOV, SIMV, Chikungunya virus, Simbu virus, Okola virus, Bunyamwere virus, Avian malaria parasites (*Plasmodium* spp.) | *Er. chrysogaster*, *Er. inornatus*, *Er. oedipodeios*, *Er. leucopous*, *Er. grahami* | Repellents |
| **Inornatus group**  *Er. argyrurus*, *Er. forcipulatus*, *Er. inornatus*, *Er. peniciillatus* |  |  |  |
| **Quinquivittatus group**  *Er. dracaenae*, *Er. quinquevittatus*, *Er. tonsus* |  |  |  |
| **Oedipodeios group**  *Er. adami*, *Er. caillardi*, *Er. eouzani*, *Er. oedipodeios*, *Er. rickenbachi*, *Er. salauni*, *Er. wansoni* |  |  |  |
| **Pliolecus grou**p  *Er. ferrarai,* *Er. germaini*, *Er. lacani*, *Er. plioleucus plioleucus*, *Er. ravissei* |  |  |  |
| **Leucopous group**  *Er. brenguesi*, *Er. jani*, *Er. leucopous*, *Er. productus* |  |  |  |

Abbreviations: SPOV: Spondweni, NKOV: Nkolbisson virus, SIMV: Semlinki virus, MIDV: Middleburg virus; NTAV: Ntaya virus.

**Additional file 5: Table S5.** *Coquillettidia* mosquitoes (eight species), pathogens transmitted and control interventions in Cameroon.

| **Species** | **Pathogens** | **Main vectors species in Cameroon** | **Vector control measures** |
| --- | --- | --- | --- |
| *Cq. fraseri*, *Cq. pseudoconopas*, *Cq. metallica*, *Cq. aurites*, *Cq. maculipennis*, *Cq. annetii*, *Cq. versicolor*, *Cq. cristata* | Avian malaria parasites | *Cq. maculipennis*, *Cq. pseudoconopas*, *Cq. aurites* | Removal of vegetation in rivers, repellents |

**Additional file 5: Table S6**. *Mansonia* mosquitoes (two species), pathogens transmitted and control interventions in Cameroon.

| **Species** | **Pathogens** | **Vector species in Cameroon** | **Vector control measures** |
| --- | --- | --- | --- |
| *Ma. africana*, *Ma. uniformis* | Avian malaria parasites | *Ma. uniformis* | IRS, LLINs, coils, repellents, removal of vegetation |

**Additional file 5: Table S7**. Mosquito species (*n* = 62) of unknown or less medical and veterinary importance: species composition, pathogens transmitted and control interventions in Cameroon. Species are grouped by genera.

| **Species** |
| --- |
| ***Toxorhynchites***  *Tx. kaimosi*, *Tx. viridibasis*, *Tx. brevipalpis*, *Tx. barbipes*, *Tx. camaronis*, *Tx. evansae*, *Tx. phytophagus*, *Tx. rickenbachi* |
| ***Uranotaenia***  *Ur. annulata*, *Ur. apicotaeniata*, *Ur. cavernicola*, *Ur. fusca*, *Ur. henriquei*, *Ur. mashonaensis*, *Ur. musarum*, *Ur. nigripes*, *Ur. nigromaculata*, *Ur. nivipous*, *Ur. ornata*, *Ur. ototomo*, *Ur. rickenbachi*, *Ur. shillitonis*, *Ur. yovani*, *Ur. alba*, *Ur. alboabdominalis*, *Ur. balfouri*, *Ur. bilineata*, *Ur. caeruleocephala*, *Ur. caliginosa*, *Ur. chorleyi*, *Ur. connali*, *Ur. fraseri*, *Ur. mayeri*, *Ur. pallidocephala*, *Ur. philonuxia* |
| ***Mimomyia***  *Mi. mediolineata*, *Mi. xanthozona*, *Mi. flavopicta*, *Mi. hispida*, *Mi. lacustris*, *Mi. mimomyiaformis*, *Mi. pallida*, *Mi. perplexens*, *Mi. plumosa* |
| ***Malaya***  *Ml. farquharsoni*, *Ml. fraseri*, *Ml. marceli*, *Ml. moucheti*, *Ml. taeniarostris*, *Ml. trichorostris* |
| ***Hodgesia***  *Ho. cyptopus*, *Ho. psectropus*, *Ho. nigeriae* |
| ***Ficalbia***  *Fi. malfeyti*, *Fi. uniformis* |
| ***Orthopodomyia***  *Or. aureoantennata*, *Or. nkolbissonensis*, *Or. ototomoensis* |
| ***Aedeomyia***  *Ad. africana*, *Ad. furfurea* |
| ***Culiseta***  *Cs. fraseri* |
| ***Lutzia***  *Lut. tigripes* |
